# Nutritional-inflammatory-metabolic indices associated with in-hospital mortality in acute kidney injury patients undergoing continuous renal replacement therapy: dose–response analysis and machine learning-based risk stratification

**DOI:** 10.3389/fnut.2026.1850255

**Published:** 2026-06-29

**Authors:** Yingying Cao, Guangxin Gu, Ruiwen Wang, Jiuxu Bai, Zhe Hao, Bin Wang, Ting Yang, Yu Wang, Yan Zhang

**Affiliations:** 1 Department of Blood Purification, General Hospital of Northern Theater Command, Shenyang, China; 2 Key Laboratory of Environmental Stress and Chronic Disease Control & Prevention, Ministry of Education, China Medical University, Shenyang, China; 3 Department of Epidemiology, School of Public Health, China Medical University, Shenyang, China; 4 Department of Orthopedics, General Hospital of Northern Theater Command, Shenyang, China

**Keywords:** acute kidney injury, Albumin-to-Alkaline Phosphatase Ratio (AAPR), continuous renal replacement therapy (CRRT), dose–response analysis, in-hospital mortality, machine learning

## Abstract

**Background:**

Composite indices derived from routine laboratory parameters can reflect nutritional, inflammatory, and metabolic status, yet their prognostic value in acute kidney injury (AKI) patients receiving continuous renal replacement therapy (CRRT) has not been systematically compared. This study aimed to evaluate and compare the prognostic value of six such indices and to integrate them into machine learning-based prediction models.

**Methods:**

This retrospective cohort study enrolled 1,732 AKI patients who received CRRT between 2020 and 2025. Six composite indices—Albumin-to-Alkaline Phosphatase Ratio (AAPR), Prognostic Nutritional Index (PNI), Albumin-Bilirubin Score (ALBI), Blood Urea Nitrogen to Creatinine Ratio (BUN/Cr), Systemic Immune-Inflammation Index (SII), and Neutrophil-to-Lymphocyte Ratio (NLR)—were calculated from laboratory data within the first 24 h of ICU admission. Associations with in-hospital mortality were evaluated using multivariable Cox regression, restricted cubic spline (RCS) analysis, and subgroup analyses. Feature selection and six machine learning models with SHAP interpretability analysis were employed for risk prediction.

**Results:**

A total of 507 patients (29.3%) died during hospitalization. AAPR was the only index with a consistent protective association in the fully adjusted model (Q4 vs. Q1: HR 0.683, 95% CI 0.524–0.890; *P* for trend = 0.007). RCS analysis revealed an L-shaped relationship (*P* for non-linearity = 0.002) with an inflection point at 0.203; below this threshold, mortality risk decreased steeply with increasing AAPR, whereas above it the risk plateaued. PNI exhibited a U-shaped association (inflection point: 33.8). ALBI and BUN/Cr lost significance after full adjustment; SII and NLR showed no stable prognostic value. Subgroup analysis confirmed the robustness of AAPR, with age as the only significant effect modifier (*P* for interaction = 0.002). Gradient Boosting achieved the highest test-set AUC (0.728), and SHAP ranked AAPR as the most important derived index.

**Conclusion:**

Among six nutritional-inflammatory-metabolic composite indices, AAPR showed the most robust prognostic value, with an L-shaped dose–response relationship identifying 0.203 as a risk stratification threshold. The U-shaped association of PNI cautions against equating higher values with better prognosis.

## Introduction

Acute kidney injury (AKI) is a major global health burden, with a pooled incidence of approximately 21.6% among hospitalized adults and an associated mortality of 23.9% (1). Beyond the acute episode, AKI is independently associated with prolonged hospitalization, progression to chronic kidney disease, and a substantial increase in both short- and long-term mortality (2). This burden is greatest in the critical care setting, where AKI affects up to 50% of critically ill adult patients (3). Among these, roughly 15% require renal replacement therapy (RRT) (4), and patients receiving continuous renal replacement therapy (CRRT) exhibit particularly poor outcomes, with short-term mortality exceeding 50% (5). Therefore, early prediction of survival outcomes in AKI patients undergoing CRRT is of paramount importance for the timely identification of high-risk individuals.

Conventional critical care prognostic tools, such as the Acute Physiology and Chronic Health Evaluation II (APACHE II) and Sequential Organ Failure Assessment (SOFA) scores, were primarily designed to capture acute physiological derangements and organ dysfunction (6, 7). However, these systems lack systematic evaluation of host nutritional reserves, immune function, and metabolic status—dimensions whose importance in determining outcomes among critically ill patients has become increasingly recognized. In particular, malnutrition and hypoalbuminemia are highly prevalent among AKI patients receiving CRRT and have been associated with impaired immune function, prolonged organ recovery, and increased mortality (8, 9). These considerations have prompted growing interest in composite indices derived from routine laboratory parameters, which are readily calculable without additional testing costs and can comprehensively reflect a patient’s pathophysiological status across nutritional, immunological, inflammatory, and metabolic dimensions. Representative indices include the Prognostic Nutritional Index (PNI), Albumin-to-Alkaline Phosphatase Ratio (AAPR), Albumin-Bilirubin Score (ALBI), Systemic Immune-Inflammation Index (SII), Neutrophil-to-Lymphocyte Ratio (NLR), and Blood Urea Nitrogen to Creatinine Ratio (BUN/Cr). Previous studies have demonstrated that these indices are independently associated with adverse outcomes in conditions such as sepsis, traumatic brain injury, and malignancies (10–12). However, studies systematically applying these indices to predict in-hospital mortality in AKI-CRRT patients remain limited.

Machine learning (ML) methods have demonstrated increasing potential in clinical prognostic prediction. Compared with traditional statistical models, ML algorithms can automatically capture complex nonlinear relationships and interaction effects among high-dimensional variables, offering inherent advantages in handling the heterogeneity of clinical data (13). Although ensemble learning algorithms such as XGBoost and random forest have shown superior discriminative performance over traditional scoring systems in AKI-CRRT mortality prediction (14), prior studies have predominantly relied on raw laboratory parameters and organ function scores as predictors, without systematically incorporating nutrition-inflammation-metabolism composite indices (15). Integrating derived indices with well-established pathophysiological relevance into ML models has the potential to enhance both predictive accuracy and clinical interpretability.

Based on the aforementioned research gaps, this study aimed to: (1) systematically evaluate the associations between six composite indices spanning nutritional, inflammatory, and metabolic dimensions and in-hospital mortality among AKI patients undergoing CRRT, and identify the index with the strongest and most robust prognostic performance; and (2) integrate the identified prognostic indices with clinical and laboratory parameters to construct and compare ML-based prediction models for in-hospital mortality. This study aspires to provide clinicians with an early risk stratification tool based on routinely available laboratory parameters, facilitating individualized decision-making for patients receiving CRRT.

## Methods

### Study design and data source

This was a single-center retrospective cohort study conducted at the General Hospital of Northern Theater Command, China. Critically ill adult patients with AKI who received CRRT in the ICU between January 1, 2020 and December 31, 2025 were screened. After applying the inclusion and exclusion criteria detailed below, a total of 1,732 patients were enrolled in the final analysis. The detailed screening process is illustrated in Supplementary Figure S1.

### Inclusion criteria

Patients were eligible for inclusion if they met all of the following criteria: (1) a confirmed diagnosis of AKI according to the 2012 Kidney Disease: Improving Global Outcomes (KDIGO) guidelines, defined as an increase in Cr ≥ 0.3 mg/dL within 48 h, or an increase in Cr to ≥1.5 times the baseline value within 7 days, or a urine output <0.5 mL/kg/h for more than 6 h, confirmed through comprehensive laboratory and clinical evaluation; (2) initiation of CRRT during hospitalization for AKI or AKI-related indications, with a treatment duration ≥24 h; (3) age ≥18 years; (4) ICU length of stay ≥24 h; (5) availability of complete laboratory data within the first 24 h of ICU admission required for the calculation of the study’s derived indices, including serum albumin (ALB), alkaline phosphatase (ALP), total bilirubin (TBIL), blood urea nitrogen (BUN), serum creatinine (Cr), platelet count (PLT), neutrophil count (NEUT), and lymphocyte count (LYMPH); and (6) for patients with multiple ICU admissions, only the first admission meeting the above criteria was included.

### Exclusion criteria

Patients were excluded if they met any of the following criteria: (1) a prior diagnosis of chronic kidney disease (CKD) stage 3b–5 or receipt of maintenance RRT before admission; (2) a history of kidney transplantation; (3) CRRT initiated for non-AKI indications (e.g., acute poisoning or isolated fluid overload without an AKI diagnosis); (4) active malignancy under palliative care, or a documented life expectancy of less than 3 months in the clinical records; (5) a confirmed diagnosis of AKI with RRT already initiated prior to ICU admission; or (6) cardiac arrest occurring before ICU admission, or withdrawal of active treatment, discharge against medical advice, or death within the first 24 h of ICU admission.

### Outcome definition

The primary outcome was in-hospital all-cause mortality. Data were obtained from the electronic medical record system, which permitted accurate ascertainment of events occurring during hospitalization but did not support post-discharge follow-up; therefore, in-hospital mortality represented the most clearly defined and reliably observed endpoint within the data structure of this study. Survival time was defined as the number of hospital days (length of stay, LOS) from ICU admission to in-hospital death or alive discharge, with patients discharged alive censored on the date of discharge. To limit the potential influence of extreme LOS values on the Cox proportional hazards model, administrative censoring was applied at day 90, following the approach described by Capuzzo et al. (16) in a European multicenter ICU cohort study; patients who remained hospitalized beyond 90 days were censored at day 90 and contributed complete risk-set information up to the point of censoring.

### Data collection and calculation of derived indices

Demographic and clinical data at admission were collected, including age, sex, surgery, mechanical ventilation (MV), extracorporeal membrane oxygenation (ECMO), septic shock, multiple organ dysfunction syndrome (MODS), diabetes, heart disease, hypertension, vasopressor use, and diuretic use. Laboratory test results obtained within the first 24 h of ICU admission were also collected, including complete blood count parameters (white blood cell count [WBC], NEUT, LYMPH, monocyte absolute count [MONO], PLT, and hemoglobin [HGB]), liver function tests (ALB, total protein [TP], ALP, alanine aminotransferase [ALT], aspartate aminotransferase [AST], and TBIL), renal function tests (BUN and Cr), coagulation parameters (international normalized ratio [INR], activated partial thromboplastin time [APTT], and fibrinogen [FIB]), and cardiac enzyme markers (creatine kinase-MB [CKMB] and lactate dehydrogenase [LDH]).

Based on published literature and clinical practice, six composite indices encompassing nutritional, inflammatory, and metabolic dimensions were calculated as the core independent variables: (1) AAPR = ALB (g/L) / ALP (U/L), reflecting nutritional status and hepatobiliary function (17); (2) PNI = ALB (g/L) + 5 × LYMPH (×10^9^/L), assessing immuno-nutritional status (18); (3) ALBI score = log_10_ [TBIL (μmol/L)] × 0.66 + ALB (g/L) × (−0.085), reflecting hepatic functional reserve (19); (4) BUN/Cr = BUN (mg/dL) / Cr (mg/dL), where BUN was converted from mmol/L to mg/dL (×2.801) and Cr from μmol/L to mg/dL (÷88.4), reflecting the differentiation between prerenal and intrinsic renal injury as well as catabolic status (20); (5) SII = PLT (×10^9^/L) × NEUT (×10^9^/L) / LYMPH (×10^9^/L), reflecting host immune-inflammatory balance (21); and (6) NLR = NEUT (×10^9^/L) / LYMPH (×10^9^/L), reflecting systemic inflammatory response (21).

### Missing data and outlier management

Missing rates were assessed for all continuous laboratory variables; variables with a missing rate exceeding 30% were excluded. For variables with a missing rate ≤30%, imputation was performed using an iterative imputer with a random forest estimator (n_estimators = 100, max_depth = 10, max_iter = 10), which better preserves nonlinear relationships and interaction effects among variables compared with conventional mean or median imputation. Outliers were identified for all continuous variables using the 1.5 × IQR rule; extreme values confirmed not to be data entry errors upon verification were subjected to bilateral Winsorization, constraining them within the 1st to 99th percentile range to reduce the influence of extreme values on model estimation stability while retaining sample information. Derived indices were calculated after completion of imputation and outlier treatment, ensuring that the underlying raw variables were complete and stable prior to calculation.

### Statistical analysis

#### Baseline characteristics

Patients were divided into a survivor group and a non-survivor group based on in-hospital mortality outcome. Continuous variables were assessed for normality using the Shapiro–Wilk test; normally distributed variables were expressed as mean ± standard deviation (SD) with between-group comparisons performed using the independent samples t-test, whereas non-normally distributed variables were expressed as median (interquartile range) with between-group comparisons performed using the Mann–Whitney U test. Categorical variables were expressed as frequencies (percentages), and between-group comparisons were performed using the chi-square test.

#### Kaplan–Meier survival analysis

Each of the six derived indices was categorized into quartiles (Q1 [lowest] to Q4 [highest]), and Kaplan–Meier survival curves were plotted with between-group differences assessed using the log-rank test. Number-at-risk tables were displayed beneath the plots.

#### Cox proportional hazards regression analysis

Univariate Cox regression was used to screen clinical covariates significantly associated with in-hospital mortality. Based on these results, three progressively adjusted Cox proportional hazards regression models were constructed to evaluate the association of each derived index with in-hospital mortality: Model 1, unadjusted; Model 2, adjusted for sex, with age included as a stratification variable; and Model 3, further adjusted for clinically significant variables identified in univariate analysis (MV, ECMO, septic shock, MODS, hypertension, and diuretic use), with age, vasopressor use, and surgery included as stratification variables. Results were reported as hazard ratios (HRs) with 95% confidence intervals (CIs). Trend tests were conducted by entering the grouped variable as a continuous variable in the model.

The proportional hazards (PH) assumption was assessed using Schoenfeld residual tests. In the initial models with all variables entered as covariates, age, vasopressor use, and surgery violated the PH assumption (all *p* < 0.05; Supplementary Tables S2–S7). These variables were therefore modeled as stratification rather than covariates—age in Models 2 and 3, and vasopressor use and surgery additionally in Model 3—allowing a separate baseline hazard for each stratum (age dichotomized as <65 vs. ≥65 years). The PH assumption was then re-assessed in the stratified models (Table S8). The same stratification was applied to the RCS and threshold-effect models.

#### Dose–response analysis and subgroup analysis

To explore dose–response relationships and potential nonlinear associations between each derived index and in-hospital mortality risk, restricted cubic spline (RCS) analysis was performed within Cox proportional hazards models, adjusted for the Model 3 covariates, with age, vasopressor use, and surgery handled by stratification (as in Model 3). Four knots were placed at the 5th, 35th, 65th, and 95th percentiles as recommended by Harrell, with the median of each index serving as the reference value. The x-axis range was restricted to the 2.5th to 97.5th percentiles to avoid the influence of extreme values. The overall association was evaluated using a joint Wald test (*P* for overall), and the nonlinear association was assessed using a joint Wald test for the nonlinear spline terms (*P* for non-linearity). For indices with a significant nonlinear test (*p* < 0.05), threshold effect analysis was further conducted using a two-piecewise linear Cox model. Candidate inflection points from the 10th to the 90th percentile were evaluated, with the optimal threshold determined by minimizing the Akaike information criterion (AIC). HRs, 95% CIs, and *p* values were reported separately for each side of the inflection point.

Stratified analyses were performed across the 10 subgroups defined by the Model 3 covariates, including age (<65 vs. ≥65 years), sex, surgery, MV, ECMO, septic shock, MODS, hypertension, vasopressor use, and diuretic use. The dichotomization strategy for each derived index was adaptively determined based on the RCS results: for indices with a significant nonlinear test, the optimal inflection point from the threshold effect analysis was used as the cutoff; for indices with a linear association, the median was used. Within each subgroup, the low-value group served as the reference, unadjusted Cox models were used to estimate HRs (95% CIs), and effect modification was assessed using a multiplicative interaction term (*P* for interaction).

### Feature selection

To construct ML prediction models, the following feature selection procedure was employed. Candidate independent variables comprised 12 baseline clinical variables, 6 derived indices, and laboratory parameters without collinearity with the derived indices (WBC, MONO, HGB, TP, ALT, AST, INR, APTT, FIB, CKMB, and LDH). Raw component variables used to calculate the derived indices (NEUT, LYMPH, PLT, ALB, ALP, TBIL, BUN, and Cr) were excluded due to inherent collinearity.

1. Dataset partitioning: The data were split into training and test sets at a 7:3 ratio using stratified random sampling, with in-hospital mortality as the stratification variable to ensure consistent event rates between the two sets. All subsequent feature selection steps were performed exclusively on the training set; the test set was not involved in any selection process to prevent data leakage.
2. Collinearity screening: Spearman rank correlation coefficient matrices were computed for all independent variables in the training set. For highly correlated variable pairs (|*r*| ≥ 0.6), the variable with a stronger correlation with the outcome was retained. When a highly correlated pair included a derived index and the difference in outcome correlations was less than 0.05, the derived index was preferentially retained.
3. Boruta feature selection: The Boruta algorithm (R package Boruta) was applied to the training set after collinearity screening, with a maximum of 100 iterations and 1,000 decision trees per iteration (num.trees = 1,000). Variables classified as “Tentative” were resolved using the TentativeRoughFix function, and only variables classified as “Confirmed” were retained (22).

### ML model construction and evaluation

Based on the selected features, six ML classification models were constructed to predict in-hospital mortality in AKI-CRRT patients: RF, XGBoost, LightGBM, GB, SVM, and LR. Continuous variables were standardized using min-max normalization (MinMaxScaler), with normalization parameters fitted on the training set only and then applied to the test set to prevent data leakage. Binary variables were retained in their original encoding. Given that in-hospital mortality was a low-frequency event, oversampling techniques (e.g., SMOTE) were not employed to avoid overfitting caused by synthetic samples. Class imbalance was addressed through internal class weight adjustment mechanisms within each model. All models were tuned using Bayesian optimization (scikit-optimize BayesSearchCV) with stratified 5-fold cross-validation (StratifiedKFold) and ROC-AUC as the optimization objective. To control model complexity and overfitting risk, strong regularization constraints were applied to tree-based models. The optimal classification threshold for each model was determined on the training set by maximizing the Youden index across a range of threshold values.

Model performance on the test set was evaluated in terms of discrimination, classification, calibration, and clinical utility. Discriminative ability was assessed using AUC-ROC, with between-model comparisons performed using the DeLong test. The precision-recall curve and average precision (AP) were additionally reported given the class imbalance in the outcome. Classification metrics included sensitivity, specificity, precision, NPV, F1 score, MCC, and Youden index (23). Calibration was evaluated using calibration curves with Platt scaling, Brier scores, and the Hosmer-Lemeshow goodness-of-fit test. Clinical utility was assessed via DCA, with integrated net benefit calculated across a range of clinically relevant threshold probabilities (24). All performance metrics were computed as means with 95% CIs via bootstrap resampling (1,000 iterations). For the best-performing model, SHAP analysis was conducted for both global and local interpretability.

### Software and statistical standards

Data preprocessing, derived index calculation, Cox regression, RCS analysis, Kaplan–Meier survival analysis, and ML modeling were performed using Python 3.9.12, with primary libraries including pandas, numpy, lifelines, scikit-learn, XGBoost, LightGBM, and scikit-optimize. Feature selection was conducted in R 4.5.1 using the Boruta and corrplot packages. All tests were two-sided, and *p* < 0.05 was considered statistically significant.

## Results

### Baseline characteristics

A total of 1,732 AKI patients who received CRRT were enrolled, of whom 507 (29.3%) died during hospitalization and 1,225 (70.7%) were discharged alive. The median age was 59 years, 70.7% were male, and the median LOS was 15 days. The non-survivor group was older (*p* < 0.0001) and had a shorter LOS than the survivor group (*p* < 0.0001). All 507 in-hospital deaths occurred within 90 days of hospitalization (median LOS among decedents 10 days, IQR 4–20 days), with deaths concentrated in the early hospitalization period; only 28 patients (1.6%) remained hospitalized beyond 90 days, all of whom were discharged alive, indicating that these patients had survived the acute phase of illness. The non-survivor group had significantly higher proportions of MV (95.1% vs. 69.6%), septic shock (22.7% vs. 9.6%), ECMO use, and vasopressor use (all *p* < 0.01). No significant between-group differences were observed for diabetes or surgery. The non-survivor group had lower ALB and Cr levels, and higher ALP, TBIL, ALT, AST, and INR values (all *p* < 0.001). Among the derived indices, AAPR and PNI were significantly lower in the non-survivor group, whereas the ALBI score and BUN/Cr were significantly higher (all *p* < 0.0001); no significant differences were observed for SII or NLR. Detailed comparisons are presented in Table 1.

**Table 1:** Baseline demographic, clinical, and laboratory characteristics of AKI-CRRT patients stratified by in-hospital survival status.

Variable	Total (*N* = 1732)	Survivors (*N* = 1,225)	Non-survivors (*N* = 507)	*P* value
Demographics				
Age, years	59.00 [45.00–68.00]	57.00 [43.00–67.00]	62.00 [50.00–70.00]	<0.0001
Clinical characteristics				
Sex				0.5461
Female	508 (29.3%)	365 (29.8%)	143 (28.2%)	
Male	1,224 (70.7%)	860 (70.2%)	364 (71.8%)	
Surgery				0.8124
No	831 (48.0%)	585 (47.8%)	246 (48.5%)	
Yes	901 (52.0%)	640 (52.2%)	261 (51.5%)	
Mechanical ventilation				<0.0001
No	398 (23.0%)	373 (30.4%)	25 (4.9%)	
Yes	1,334 (77.0%)	852 (69.6%)	482 (95.1%)	
ECMO/IABP				<0.0001
No	1,546 (89.3%)	1,125 (91.8%)	421 (83.0%)	
Yes	186 (10.7%)	100 (8.2%)	86 (17.0%)	
Septic shock				<0.0001
No	1,500 (86.6%)	1,108 (90.4%)	392 (77.3%)	
Yes	232 (13.4%)	117 (9.6%)	115 (22.7%)	
MODS				0.0041
No	1,512 (87.3%)	1,088 (88.8%)	424 (83.6%)	
Yes	220 (12.7%)	137 (11.2%)	83 (16.4%)	
Diabetes				0.4510
No	1,334 (77.0%)	937 (76.5%)	397 (78.3%)	
Yes	398 (23.0%)	288 (23.5%)	110 (21.7%)	
Heart disease				0.0039
No	1,654 (95.5%)	1,158 (94.5%)	496 (97.8%)	
Yes	78 (4.5%)	67 (5.5%)	11 (2.2%)	
Hypertension				0.0130
No	1,103 (63.7%)	757 (61.8%)	346 (68.2%)	
Yes	629 (36.3%)	468 (38.2%)	161 (31.8%)	
Vasopressor use				<0.0001
No	1,024 (59.1%)	768 (62.7%)	256 (50.5%)	
Yes	708 (40.9%)	457 (37.3%)	251 (49.5%)	
Diuretic use				0.0509
No	640 (37.0%)	471 (38.4%)	169 (33.3%)	
Yes	1,092 (63.0%)	754 (61.6%)	338 (66.7%)	
Laboratory parameters (derived index components)				
Albumin, g/L	31.18 ± 6.61	31.61 ± 6.44	30.15 ± 6.91	<0.0001
Alkaline phosphatase, U/L	74.94 [57.25–99.11]	72.85 [57.00–95.94]	80.02 [59.34–109.32]	0.0002
Neutrophil count, ×10^9^/L	10.51 [7.30–14.60]	10.27 [7.31–14.03]	11.30 [7.22–15.64]	0.0207
Lymphocyte count, ×10^9^/L	0.80 [0.50–1.20]	0.80 [0.52–1.19]	0.79 [0.50–1.20]	0.3580
Platelet count, ×10^9^/L	164.00 [111.00–227.00]	169.00 [117.00–232.00]	158.66 [99.50–216.50]	0.0060
Total bilirubin, μmol/L	13.00 [7.57–22.82]	12.40 [7.10–22.06]	14.60 [8.70–26.20]	0.0003
Blood urea nitrogen, mmol/L	12.73 [7.73–20.11]	13.27 [7.85–20.72]	11.86 [7.51–19.05]	0.1012
Serum creatinine, μmol/L	180.68 [96.02–352.70]	198.75 [96.30–401.70]	154.20 [93.80–262.93]	<0.0001
Laboratory parameters (other)				
White blood cell, ×10^9^/L	12.10 [8.60–16.30]	12.00 [8.60–15.80]	12.90 [8.70–17.10]	0.0239
Monocyte count, ×10^9^/L	0.55 [0.33–0.81]	0.55 [0.33–0.80]	0.57 [0.33–0.86]	0.8145
Hemoglobin, g/L	114.00 [94.00–136.00]	113.00 [94.00–136.00]	115.00 [92.00–136.00]	0.5442
Total protein, g/L	55.30 [49.40–62.30]	55.90 [50.40–62.90]	54.10 [47.30–61.25]	<0.0001
Alanine aminotransferase, U/L	28.92 [15.50–85.68]	26.20 [14.80–79.00]	37.01 [18.29–106.47]	<0.0001
Aspartate aminotransferase, U/L	51.83 [23.02–166.89]	47.70 [21.54–151.19]	61.12 [27.90–199.99]	0.0003
INR	1.19 [1.10–1.40]	1.18 [1.09–1.35]	1.24 [1.12–1.53]	<0.0001
APTT, s	36.10 [30.19–43.70]	35.90 [29.60–42.50]	36.80 [31.05–46.30]	0.0143
Fibrinogen, g/L	4.29 [2.90–5.82]	4.38 [3.01–5.90]	4.06 [2.63–5.50]	0.0040
CK-MB, U/L	25.75 [13.00–76.91]	24.00 [12.70–72.20]	29.70 [14.92–91.33]	0.0077
Lactate dehydrogenase, U/L	426.15 [260.90–782.22]	416.00 [254.00–765.00]	444.61 [279.00–817.93]	0.0362
Nutritional-inflammatory-metabolic indices				
AAPR	0.42 [0.31–0.55]	0.43 [0.32–0.57]	0.39 [0.26–0.51]	<0.0001
PNI	35.65 [30.85–40.60]	35.90 [31.50–41.05]	34.40 [29.15–40.11]	<0.0001
ALBI score	−1.89 ± 0.62	−1.94 ± 0.61	−1.77 ± 0.64	<0.0001
BUN/Cr ratio	16.22 [11.65–22.61]	15.79 [10.88–21.89]	17.95 [13.19–24.40]	<0.0001
SII	2085.89 [1132.01–3635.03]	2093.33 [1184.54–3471.00]	2077.19 [1016.28–4026.23]	0.7520
NLR	13.29 [8.28–21.29]	13.09 [8.33–20.10]	13.98 [8.16–24.05]	0.0827
Clinical outcomes				
Length of hospital stay, days	15.00 [7.00–27.00]	17.00 [9.00–29.00]	10.00 [4.00–20.00]	<0.0001

### Association between derived indices and in-hospital mortality

**Figure 1:**
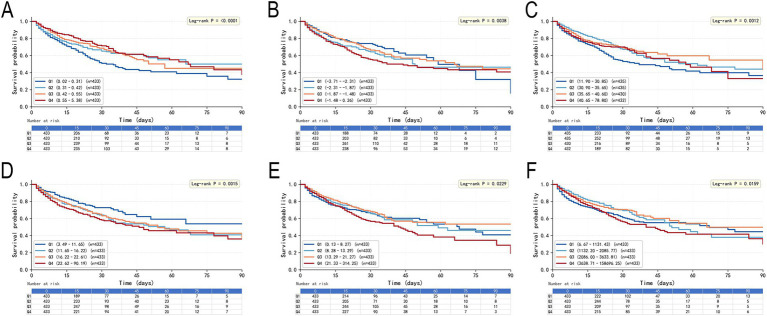
Kaplan–Meier survival curves for six derived indices stratified by quartiles. Patients (N = 1,732) were divided into quartiles (Q1–Q4) for each index; the value range and the number of patients in each quartile are shown in the inset legend of each panel. **(A)** AAPR; **(B)** ALBI score; **(C)** PNI; **(D)** BUN/Cr; **(E)** NLR; **(F)** SII. The vertical axis is the probability of in-hospital survival and the horizontal axis is the time from ICU admission in days (administratively censored at day 90). The four colored step curves denote the quartile groups from Q1 (lowest) to Q4 (highest). Between-group differences were assessed with the log-rank test, and the corresponding *p* value is given in the upper-right corner of each panel. The number-at-risk table beneath each panel reports the patients remaining at risk in each quartile at 0, 15, 30, 45, 60, 75, and 90 days.

Kaplan–Meier survival curves for all six derived indices categorized by quartiles demonstrated statistically significant between-group differences (all log-rank *p* < 0.05). AAPR (*p* < 0.0001) and PNI (*p* = 0.0012) exhibited a clear dose–response gradient, with the lowest survival in Q1 and the highest in Q4. The ALBI score (*p* = 0.0038) and BUN/Cr (*p* = 0.0015) showed an opposite trend, with the lowest survival in Q4. SII (*p* = 0.0159) and NLR (*p* = 0.0229) demonstrated significant between-group differences, but the gradient separation was less pronounced (Figure 1). Univariate Cox regression identified age, surgery, MV, ECMO, septic shock, MODS, hypertension, vasopressor use, and diuretic use as significantly associated with in-hospital mortality (all *p* < 0.05), whereas diabetes and heart disease did not reach significance. Model 2 was adjusted for sex, with age included as a stratification variable; Model 3 was further adjusted for the clinically significant variables above, with age, vasopressor use, and surgery included as stratification variables (Supplementary Table S1).

Cox regression results for the six derived indices by quartiles are presented in Table 2. In Models 2 and 3, age (and additionally vasopressor use and surgery in Model 3) was handled by stratification rather than as a covariate; hazard ratios for these variables are therefore not estimated. AAPR demonstrated a consistent protective effect across all three models, with the Q4 group showing significantly lower mortality risk compared with Q1 (Model 1: HR 0.580, 95% CI 0.453–0.741; Model 3: HR 0.683, 95% CI 0.524–0.890, *p* = 0.005; *P* for trend = 0.007). PNI showed significant protective effects in Q2 and Q3 in the unadjusted model (HR 0.656 and 0.680, respectively; both *p* < 0.01), but only Q2 remained significant in the fully adjusted model (HR 0.728, 95% CI 0.572–0.925, *p* = 0.009), and the trend test was no longer significant (*P* for trend = 0.732). The ALBI score and BUN/Cr showed significantly elevated risk in Q4 in the unadjusted and partially adjusted models, but the associations attenuated to non-significance after full adjustment. SII and NLR did not demonstrate significant dose–response trends in any model (all *P* for trend > 0.05).

**Table 2:** Cox proportional hazards regression analysis of the association between derived indices (by quartiles) and in-hospital mortality across three progressively adjusted models.

Derived index	Group	Model 1	*P*-value (M1)	Model 2	*P*-value (M2)	Model 3	*P*-value (M3)
AAPR	Q1 (0.02–0.31) (Ref)	1.000 (Ref)		1.000 (Ref)		1.000 (Ref)	
AAPR	Q2 (0.31–0.42)	0.725 (0.572–0.919)	0.0078	0.741 (0.584–0.939)	0.0132	0.872 (0.682–1.115)	0.2734
AAPR	Q3 (0.42–0.55)	0.665 (0.525–0.843)	0.0007	0.691 (0.545–0.876)	0.0023	0.869 (0.676–1.116)	0.2712
AAPR	Q4 (0.55–5.38)	0.580 (0.453–0.741)	<0.0001	0.598 (0.467–0.765)	<0.0001	0.683 (0.524–0.890)	0.0047
AAPR	P for trend		<0.0001		<0.0001		0.0073
PNI	Q1 (11.90–30.85) (Ref)	1.000 (Ref)		1.000 (Ref)		1.000 (Ref)	
PNI	Q2 (30.90–35.65)	0.656 (0.517–0.831)	0.0005	0.679 (0.535–0.860)	0.0014	0.728 (0.572–0.925)	0.0094
PNI	Q3 (35.65–40.60)	0.680 (0.534–0.867)	0.0018	0.710 (0.557–0.906)	0.0059	0.798 (0.623–1.021)	0.0729
PNI	Q4 (40.65–78.80)	0.789 (0.622–1.001)	0.0507	0.850 (0.668–1.082)	0.1872	0.970 (0.757–1.244)	0.8131
PNI	P for trend		0.0452		0.1543		0.7324
ALBI score	Q1 (−3.71–-2.31) (Ref)	1.000 (Ref)		1.000 (Ref)		1.000 (Ref)	
ALBI score	Q2 (−2.31–-1.87)	1.317 (1.008–1.721)	0.0438	1.298 (0.993–1.697)	0.0562	1.149 (0.875–1.508)	0.3174
ALBI score	Q3 (−1.87–-1.48)	1.070 (0.817–1.402)	0.6234	1.055 (0.805–1.385)	0.6966	0.961 (0.727–1.271)	0.7821
ALBI score	Q4 (−1.48–0.26)	1.500 (1.163–1.936)	0.0018	1.441 (1.115–1.862)	0.0052	1.121 (0.855–1.470)	0.4080
ALBI score	P for trend		0.0111		0.0259		0.7031
BUN/Cr	Q1 (3.49–11.65) (Ref)	1.000 (Ref)		1.000 (Ref)		1.000 (Ref)	
BUN/Cr	Q2 (11.65–16.22)	1.453 (1.103–1.915)	0.0079	1.387 (1.051–1.831)	0.0207	1.254 (0.946–1.662)	0.1156
BUN/Cr	Q3 (16.22–22.61)	1.401 (1.064–1.845)	0.0162	1.328 (1.006–1.752)	0.0454	1.157 (0.874–1.532)	0.3085
BUN/Cr	Q4 (22.62–90.19)	1.703 (1.301–2.228)	0.0001	1.536 (1.166–2.025)	0.0023	1.313 (0.992–1.738)	0.0570
BUN/Cr	P for trend		0.0004		0.0072		0.1279
SII	Q1 (6.67–1131.43) (Ref)	1.000 (Ref)		1.000 (Ref)		1.000 (Ref)	
SII	Q2 (1132.20–2085.77)	0.758 (0.591–0.972)	0.0289	0.768 (0.599–0.985)	0.0373	0.800 (0.622–1.030)	0.0835
SII	Q3 (2086.00–3633.81)	0.773 (0.601–0.993)	0.0437	0.775 (0.603–0.997)	0.0470	0.826 (0.641–1.066)	0.1422
SII	Q4 (3638.71–158696.25)	1.038 (0.825–1.307)	0.7490	1.058 (0.840–1.332)	0.6329	1.044 (0.826–1.320)	0.7165
SII	P for trend		0.7200		0.6299		0.6692
NLR	Q1 (0.13–8.27) (Ref)	1.000 (Ref)		1.000 (Ref)		1.000 (Ref)	
NLR	Q2 (8.28–13.29)	0.912 (0.707–1.176)	0.4784	0.904 (0.700–1.166)	0.4359	0.851 (0.658–1.102)	0.2213
NLR	Q3 (13.29–21.27)	0.823 (0.640–1.059)	0.1300	0.821 (0.638–1.056)	0.1244	0.762 (0.590–0.984)	0.0370
NLR	Q4 (21.33–314.25)	1.182 (0.935–1.494)	0.1621	1.168 (0.923–1.478)	0.1953	0.997 (0.784–1.268)	0.9807
NLR	P for trend		0.2571		0.2896		0.8590

The PH assumption was assessed using Schoenfeld residual tests. In the initial models with all variables entered as covariates, age, vasopressor use, and surgery violated the assumption (all *p* < 0.05; Supplementary Tables S2–S7); these variables were therefore handled by stratification (age in Models 2 and 3; vasopressor use and surgery additionally in Model 3). After stratification (Supplementary Tables S8), the global PH assumption was satisfied in Model 2 for five of the six indices, and—most importantly—the exposure terms (index quartiles) satisfied the PH assumption in the fully adjusted Model 3 for all six indices, indicating that the estimated associations between the indices and mortality were not affected by non-proportionality. A residual global violation persisted in Model 3 and arose solely from individual adjustment covariates (MODS and sex) rather than from the primary exposures; further stratification of these covariates was not pursued, as it produced excessively sparse strata and unstable estimates. To confirm that this residual non-proportionality did not affect the principal findings, a sensitivity analysis stratifying the follow-up at the median event time (10 days) was performed (Supplementary Table S9). The protective association of AAPR was directionally consistent in both periods (early, HR 0.76, 95% CI 0.53–1.10; late, HR 0.55, 95% CI 0.37–0.80), being more pronounced in the later period, indicating that the overall conclusion was robust to the residual time-varying effects.

### Dose–response analysis and subgroup analysis

**Figure 2:**
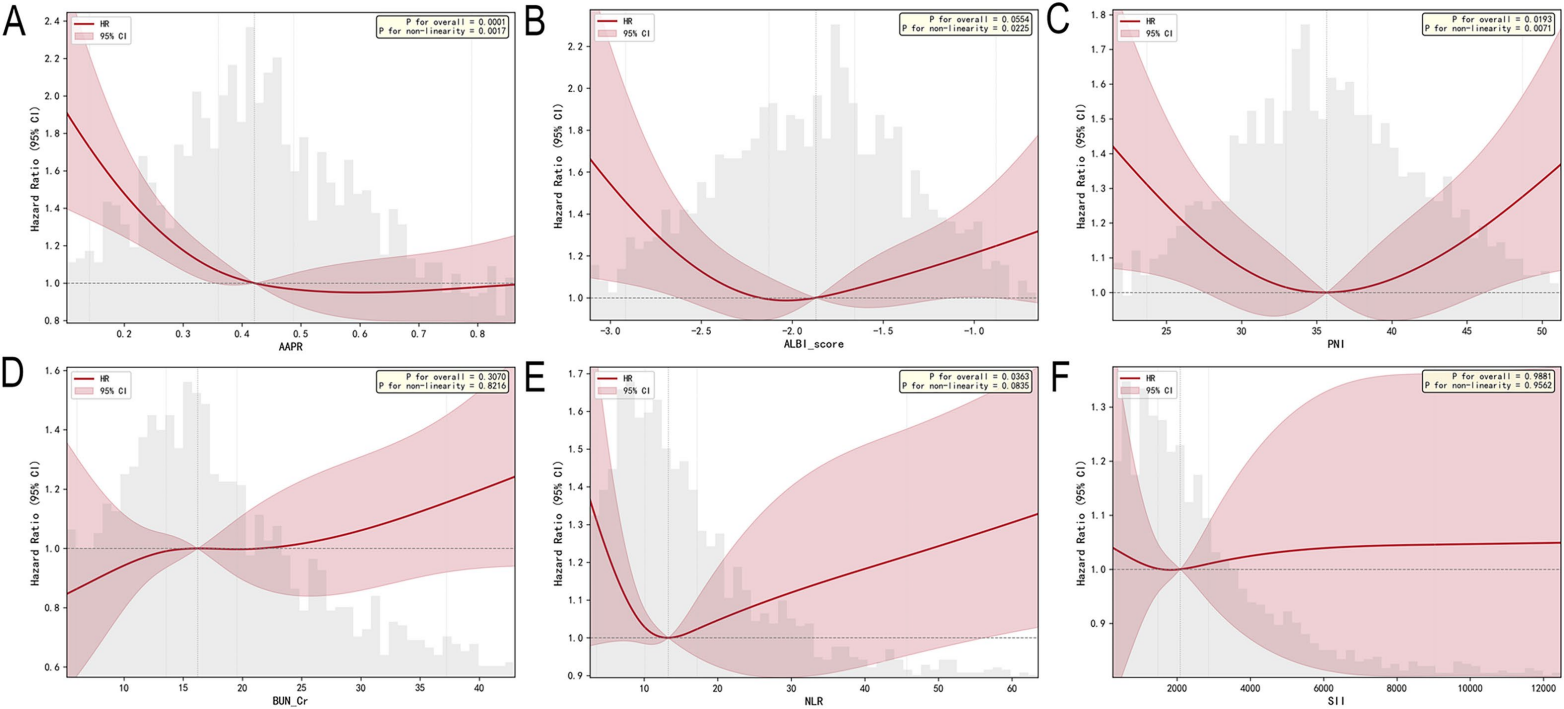
Restricted cubic spline analysis of the dose–response relationships between derived indices and in-hospital mortality. **(A)** AAPR; **(B)** ALBI score; **(C)** PNI; **(D)** BUN/Cr; **(E)** NLR; **(F)** SII. RCS curves were fitted within Cox proportional hazards models adjusted for the Model 3 covariates, with age, vasopressor use, and surgery handled by stratification. Four knots were placed at the 5th, 35th, 65th, and 95th percentiles, and the median of each index (vertical dotted reference line) served as the reference value (HR = 1). The solid red line is the estimated hazard ratio (HR) and the shaded red band the corresponding 95% confidence interval (CI); the horizontal dashed line marks HR = 1. The light-gray histogram shows the distribution of each index, and the *x*-axis is restricted to the 2.5th–97.5th percentiles to limit the influence of extreme values. The overall association (*P* for overall, joint Wald test for all spline terms) and the nonlinear association (*P* for non-linearity, joint Wald test for the nonlinear spline terms) are reported in the upper-right corner of each panel.

RCS analysis adjusted for the Model 3 covariates revealed significant nonlinear associations for AAPR (*P* for overall = 0.0001, *P* for non-linearity = 0.0017), PNI (*P* for overall = 0.0193, *P* for non-linearity = 0.0071), and the ALBI score (*P* for non-linearity = 0.0225). AAPR exhibited an L-shaped curve, with risk rising steeply in the lower range and plateauing at approximately 0.4. PNI displayed a U-shaped curve, with elevated mortality risk at both extremes. The ALBI score also demonstrated a U-shaped association. NLR showed a significant overall association (*P* for overall = 0.0363) but the nonlinear test did not reach significance (*p* = 0.0835). Neither BUN/Cr nor SII showed significant overall or nonlinear associations (Figure 2).

Threshold effect analysis using a two-piecewise linear Cox model was performed for the indices with significant nonlinearity. The optimal inflection point for AAPR was 0.203; below this point, mortality risk decreased steeply with increasing AAPR, whereas above it the risk plateaued (HR 0.797, 95% CI 0.475–1.340, *p* = 0.393). The optimal inflection point for PNI was 33.8, with opposite effect directions on either side: increases below the inflection point were protective (HR 0.966, *p* = 0.004), while increases above it were associated with elevated risk (HR 1.022, *p* = 0.013). The optimal inflection point for the ALBI score was −2.70; increases below this point were significantly associated with reduced mortality risk (HR 0.122, *p* < 0.0001), while the upward trend above this point did not reach significance (HR 1.178, *p* = 0.059). HRs and 95% CIs for both sides of each inflection point are detailed in Supplementary Table S10.

Subgroup analysis results are presented in Supplementary Table S11. The protective effect of AAPR was the most robust, reaching significance in the majority of subgroups, with non-significant associations only in the hypertension (HR 0.74, *p* = 0.347), surgery (HR 0.73, *p* = 0.186), and septic shock (HR 0.66, *p* = 0.051) subgroups. Age was the only significant effect modifier (*P* for interaction = 0.002), with a stronger protective effect in patients aged <65 years (HR 0.41 vs. 0.69). For PNI, significant effect modification was observed in the hypertension subgroup (*P* for interaction < 0.001): higher PNI was protective among patients without hypertension (HR 0.68, *p* < 0.001) but associated with increased risk among those with hypertension (HR 1.50, *p* = 0.025). BUN/Cr showed a significant interaction in the MODS subgroup (*P* for interaction = 0.048), with elevated mortality risk in the high BUN/Cr group among patients with MODS (HR 2.00, *p* = 0.003). SII, NLR, and the ALBI score did not demonstrate significant or consistent associations across subgroups, nor significant interaction effects.

### ML model construction and evaluation

**Figure 3:**
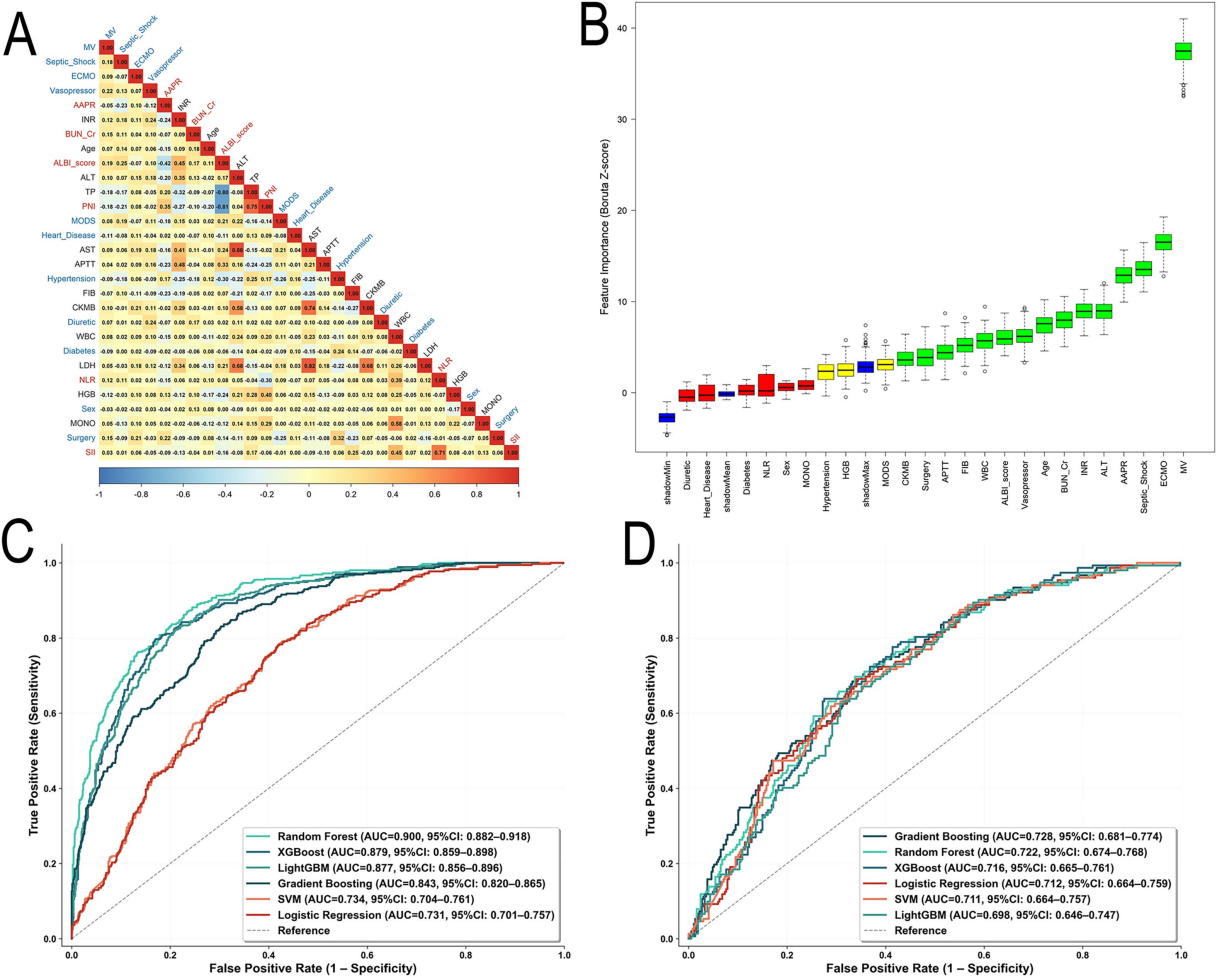
Feature selection and model discrimination. **(A)** Spearman rank correlation matrix of the candidate variables; the color scale (blue to red) and the value in each cell indicate the correlation coefficient (−1 to +1). **(B)** Boruta feature-importance ranking; each box plot shows the distribution of the importance (Z-score) across iterations for one variable. Green boxes denote confirmed (important) features, yellow boxes tentative features, and red boxes rejected features; the blue boxes (shadowMin, shadowMean, shadowMax) are the shadow features that define the decision threshold. **(C,D)** Receiver operating characteristic (ROC) curves of the six machine-learning models on the training set **(C)** and the test set **(D)**. The area under the curve (AUC) and its bootstrap 95% CI (1,000 resamples) are given for each model in the inset legend; the grey diagonal is the reference line (AUC = 0.50).

The Spearman correlation matrix on the training set identified 9 highly correlated variable pairs (|*r*| ≥ 0.6); after stepwise comparison, PNI, SII, AST, LDH, and TP were removed, and 24 variables entered Boruta selection. The Boruta algorithm confirmed 16 important features and rejected 8 (HGB, hypertension, MONO, NLR, sex, diabetes, heart disease, and diuretic use) (Figure 3A). The final 16 features comprised 6 binary variables (MV, ECMO, septic shock, vasopressor use, surgery, and MODS) and 10 continuous variables (AAPR, ALBI score, BUN/Cr, age, ALT, INR, WBC, FIB, APTT, and CKMB) (Figure 3B). Among the six derived indices, AAPR (Boruta Z-score 12.9, ranked 4th), the ALBI, and BUN/Cr passed the dual screening; PNI and SII were excluded during collinearity screening, and NLR was classified as unimportant by Boruta.

**Figure 4:**
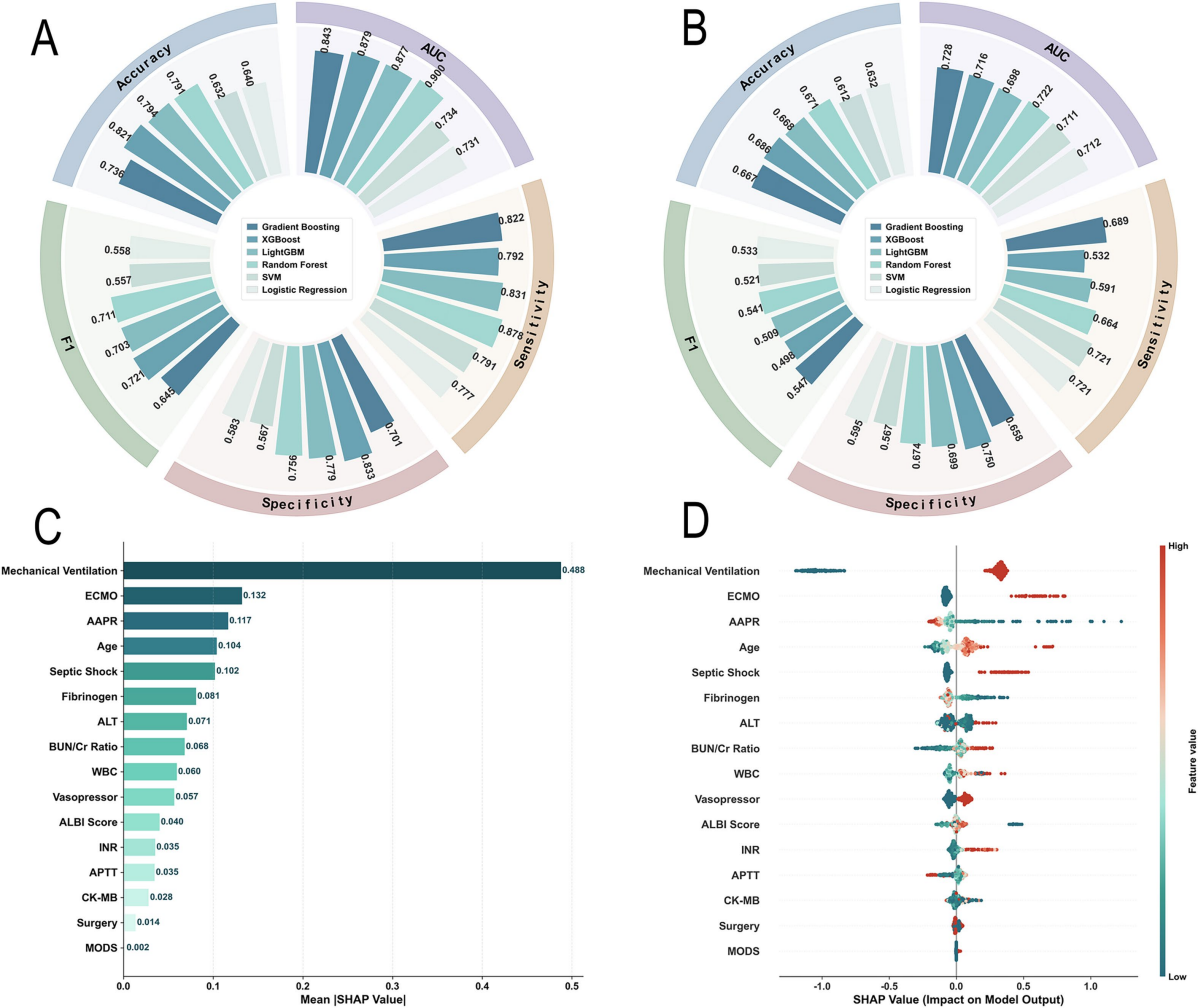
Model performance comparison and SHAP interpretability analysis. **(A,B)** Radar charts comparing the six models across five metrics—accuracy, AUC, sensitivity, specificity, and F1 score—on the training set **(A)** and the test set **(B)**; each model corresponds to one color as indicated in the central legend, and the numeric value of every metric is labeled on the corresponding bar. **(C)** Global SHAP feature importance for the optimal Gradient Boosting model, expressed as the mean absolute SHAp value (mean |SHAp value|) across all test-set patients; larger values indicate a greater average contribution to the predicted mortality risk. **(D)** SHAP summary beeswarm plot; each dot represents one patient, its horizontal position the SHAp value (impact on the model output, with positive values pushing the prediction toward death) and its color the magnitude of the feature value (red = high, blue = low). Features in **(C,D)** are ordered from top to bottom by decreasing importance.

Six ML models were constructed based on the 16 selected features. On the test set, GB achieved the highest AUC (0.728, 95% CI 0.681–0.774), followed by RF (0.722), XGBoost (0.716), LR (0.712), SVM (0.711), and LightGBM (0.698) (Supplementary Table S12 and Figures 3D, 4B). The DeLong test indicated that GB differed significantly only from LightGBM (*p* = 0.025); differences with all other models were not significant (all *p* > 0.05) (Supplementary Table S14). GB also achieved the highest AP (0.499), Youden index (0.347), and MCC (0.318), with a balanced sensitivity-specificity trade-off (0.691 vs. 0.657) (Supplementary Figure S2). Among all models, LR and SVM exhibited the most stable generalization (training-test AUC *Δ* = 0.019 and 0.023, respectively) and optimal calibration after Platt scaling (Supplementary Tables S12, S13, Figures 3C, 4A, B). GB showed the least overfitting among tree-based models (Δ = 0.115) and achieved the highest integrated net benefit on DCA (0.064), remaining the only model with positive net benefit at the 50% threshold (Supplementary Figure S2). Considering discrimination, classification performance, generalization stability, calibration, and clinical utility in aggregate, GB was selected as the optimal model.

### SHAP interpretability analysis

SHAP analysis of the optimal GB model showed that MV had the highest mean |SHAP value| (0.488), followed by ECMO (0.132), AAPR (0.117), age (0.104), and septic shock (0.102). AAPR ranked 3rd among all features and was the highest-ranked derived index (Figure 4C).

The beeswarm plot (Figure 4D) revealed that the presence of MV, ECMO, and septic shock drove predictions toward mortality, whereas AAPR displayed a clear protective pattern, with low values corresponding to large positive SHAP values and high values corresponding to negative SHAP values. The scatter plot (Supplementary Figure S3) showed that SHAP values for AAPR rose steeply in the lower range (<0.2), consistent with the L-shaped dose–response relationship identified in the RCS analysis. Interaction analysis (Supplementary Figure S4) indicated that the risk contribution of MV was further amplified in the presence of ECMO or low AAPR, while septic shock attenuated the protective effect of AAPR.

Waterfall plots (Supplementary Figure S5) illustrated three representative patient profiles: in a low-risk patient (f(x) = −3.172), the absence of MV (−1.08) and a higher AAPR (0.6) were the primary protective factors; in a high-risk patient (f(x) = 1.098), an extremely low AAPR (0.1, +1.01), advanced age (86 years, +0.59), and septic shock (+0.30) were the major risk drivers; and in an intermediate-risk patient (f(x) = 0.001), the positive contributions of ECMO (+0.52) and MV (+0.29) were offset by the protective effects of ALT and AAPR, yielding a predicted probability near the classification threshold.

## Discussion

Conventional severity scores such as APACHE II and SOFA do not systematically incorporate nutritional, hepatobiliary, and metabolic dimensions increasingly recognized as important in critically ill patients. To our knowledge, this is the first study to comprehensively compare six routine laboratory-derived composite indices spanning these dimensions in the AKI-CRRT population, using both Cox-RCS analysis and ML-based SHAP validation. The central finding was that AAPR demonstrated consistent and independent prognostic associations across the fully adjusted Cox model, RCS analysis, subgroup analysis, and SHAP ranking, identifying it as the most robust prognostic marker among the six indices evaluated.

Albumin-to-alkaline phosphatase ratio exhibited an L-shaped nonlinear association with in-hospital mortality: below the inflection point (0.203), risk decreased steeply with increasing AAPR. A large retrospective analysis of 6,894 critically ill AKI patients from the MIMIC-III database identified AAPR < 0.35 as an independent predictor of mortality (25). Similarly, an independent association between APAR (the inverse of AAPR) and mortality has been observed in a cohort of 9,741 sepsis patients (26). The lower inflection point observed in the present study is consistent with the more profound hypoalbuminemia and more extensive hepatobiliary stress characteristic of the AKI-CRRT population, and provides the first quantified risk stratification threshold for this specific group. Mechanistically, decreased ALB levels are typically associated with inflammation-induced capillary permeability, reduced hepatic synthesis, and depletion of antioxidant reserves (27, 28), and in the setting of renal failure, are also linked to chronic inflammation and vascular calcification (29). Concurrently, elevated ALP may result from cholestasis, ischemic hepatic injury, and systemic inflammation (30). AAPR thus simultaneously captures changes across nutritional, hepatic, and inflammatory dimensions, which may partly explain its sustained prognostic association after multivariable adjustment. It should be noted, however, that this study did not incorporate established intensive care severity scores such as APACHE II or SOFA in the multivariable models. Because serum albumin and its derived indices are themselves integrated markers of disease severity that decline with systemic inflammation, capillary leak, and impaired hepatic synthesis (31), and have been shown to predict mortality in critically ill patients with limited sensitivity and specificity once illness severity is accounted for (32), the absence of these severity scores raises the possibility of residual confounding by overall illness severity. Consequently, the independent prognostic value of AAPR and the other derived indices observed here may have been overestimated, and the true effect size should be re-evaluated in models that explicitly adjust for validated severity scores. The plateau segment of the L-shaped curve suggests that the clinical benefit window may be concentrated among patients with low AAPR (< 0.203) rather than the entire population. In the subgroup analysis, age was the only significant effect modifier (P for interaction = 0.002), with a stronger protective effect in patients aged <65 years, possibly attributable to reduced physiological reserve and competing mortality risks in older patients (33).

Prognostic nutritional index exhibited a U-shaped association with mortality risk, with an inflection point at 33.8. A previous study enrolling 2,076 AKI-CRRT patients reported a median PNI cutoff of 27.28 (34), further supporting the generally lower PNI levels in this population. The rising risk above the inflection point warrants cautious interpretation: in the context of low ALB levels, elevated PNI is more likely driven by increases in LYMPH, rather than by a genuine improvement in nutritional status. In severe systemic inflammation, an abnormally elevated lymphocyte count does not necessarily reflect restored immune competence but may instead arise from stress-induced demargination, a dysregulated reactive lymphocytosis following the initial inflammatory insult, or a post-infectious reactive response (35). Consistent with this interpretation, lymphocyte count itself has been shown to exhibit a U-shaped association with mortality in critically ill patients, with both lymphopenia and abnormally high counts predicting worse outcomes (36); a disproportionate rise in lymphocytes may therefore inflate the PNI without conferring true prognostic benefit, partly accounting for the increased risk observed above the inflection point. Furthermore, the reversal of effect direction in the hypertension subgroup suggests that coexisting cardiovascular comorbidities may modify the prognostic significance of PNI. Clinically, PNI should not be simplistically interpreted as “the higher the better” but rather evaluated within the context of specific value ranges.

The ALBI score and BUN/Cr both lost significance after full adjustment, suggesting that their associations with prognosis may have been largely influenced by disease severity and other clinical factors. The component variables of the ALBI score—ALB and TBIL—are susceptible to multiple confounding influences under critical illness conditions (37), potentially limiting its incremental prognostic information after adjustment for severity indicators. Nevertheless, the ALBI score was retained by the Boruta algorithm, suggesting that it may still carry predictive value within nonlinear modeling frameworks. The diagnostic utility of BUN/Cr in ICU populations remains controversial (38), as elevated ratios can arise from multiple conditions including hypo-perfusion, hypercatabolic states, and gastrointestinal bleeding. In the present study, BUN/Cr was significantly associated with mortality risk only in the MODS subgroup (HR 2.00, *p* = 0.003). SII and NLR did not demonstrate stable prognostic value in this cohort. Yoon et al. (39) found in a multicenter cohort of 1,494 AKI-CRRT patients that baseline NLR at CRRT initiation did not differ significantly between survivors and non-survivors, whereas the fold change in NLR at day 5 was independently associated with mortality, consistent with the present results based on single time-point measurements. AKI-CRRT patients are generally in a state of heightened systemic inflammation, which may attenuate the discriminative capacity of static inflammatory indices. Additionally, CRRT itself may alter circulating inflammatory marker levels through membrane adsorption and mediator clearance (40), further limiting their prognostic utility. Furthermore, AKI patients requiring CRRT generally present with markedly elevated baseline inflammatory activity, which may compress the inter-individual variability of static inflammatory indices such as SII and NLR, resulting in a ceiling effect that inherently limits their discriminative capacity for mortality prediction in this population.

GB achieved a test-set AUC of 0.728, representing moderate discriminative performance. In comparable ICU mortality prediction studies, AUCs of 0.784 and 0.756 have been reported using random forest with APACHE II and SOFA scores (14) and Lasso logistic regression (41), respectively. The present result falls within a reasonable range, considering that features were intentionally restricted to routine laboratory indices. The narrow AUC range across all six models (0.698–0.728) suggests that the performance ceiling was determined by feature information content rather than model architecture. Linear models exhibited superior generalization stability compared with tree-based models (training-test AUC *Δ* = 0.019–0.023 vs. 0.115), and their interpretability and robustness should not be overlooked in clinical settings with limited sample sizes. On DCA, GB maintained positive net benefit across the 10–50% threshold range, suggesting potential for decision support, although external validation is required before broader implementation.

Albumin-to-alkaline phosphatase ratio ranked third in SHAP global feature importance (mean |SHAP| = 0.117), and the effect gradient in its lower range was consistent with the L-shaped curve identified in the RCS analysis, exemplifying the complementary validation between conventional statistics and ML. However, SHAP values quantify the marginal contribution of a feature to the prediction output, not causal effects. Lundberg et al. (42) noted that prediction models learn conditional association patterns that conflate causal effects with confounding bias, and subsequent work has further demonstrated that quantifying feature relevance via SHAP is inherently a causal problem, as standard implementations do not distinguish between observational and interventional distributions (43). Accordingly, the SHAP results should be interpreted as indicating that AAPR is the most informative derived index rather than that it exerts a causal protective effect; causal verification remains contingent upon prospective studies.

This study has several limitations. First, the single-center retrospective design limits external generalizability; differences in CRRT initiation criteria and patient composition across centers may affect the effect sizes and optimal thresholds of the derived indices. Second, the derived indices were based on single measurements at ICU admission and cannot capture dynamic changes. As dynamic monitoring of NLR has been shown to outperform single baseline measurements in prognostic accuracy (39), incorporating serial assessments of AAPR may yield additional predictive value. Future prospective studies should therefore be designed to capture the evolving pathophysiology of these patients, for example by tracking AAPR trajectories at serial time points over the first 72 h of CRRT (e.g., at 0, 24, 48, and 72 h) and by modeling these dynamic changes—such as the magnitude and direction of early AAPR change—in relation to outcomes, which may provide greater prognostic resolution than any single baseline value. Third, the use of in-hospital mortality as the endpoint may miss early post-discharge death events. Fourth, as discussed above, the absence of validated severity scores such as APACHE II and SOFA—together with the lack of CRRT prescription parameters—may have introduced residual confounding and led to partial overestimation of the independent prognostic associations observed. Fifth, PNI and SII were excluded during collinearity screening in the ML analysis, and their nonlinear contributions could not be fully assessed within the modeling framework.

## Conclusion

Among AKI patients receiving CRRT, AAPR was the most robust prognostic marker of the six derived indices evaluated, with its L-shaped relationship highlighting particular risk-alerting value in the lower range (<0.203). The U-shaped association of PNI revealed the limitations of the “higher is better” assumption in critical care nutritional assessment. All findings are associative in nature. Future efforts should prioritize multicenter external validation of the stability of the AAPR threshold, exploration of the prognostic incremental value of dynamic monitoring, and prospective evaluation of intervention strategies targeting low AAPR on outcomes in AKI-CRRT patients.

## Data Availability

The original contributions presented in the study are included in the article/Supplementary material, further inquiries can be directed to the corresponding author/s.
