# Supplementary material for: Nutritional-inflammatory-metabolic indices associated with in-hospital mortality in acute kidney injury patients undergoing continuous renal replacement therapy: dose–response analysis and machine learning-based risk stratification
Source: Front Nutr. 2026 Jun 29;13:1850255. doi: 10.3389/fnut.2026.1850255 (PMC13359152; doi:10.3389/fnut.2026.1850255)

**Table S1. Univariate Cox Proportional Hazards Regression Analysis of Baseline Clinical Variables for In-Hospital Mortality in AKI-CRRT Patients.**

|  | HR (95% CI) | P value | P<0.05 |
| --- | --- | --- | --- |
| Sex | 0.991 (0.817-1.203) | 0.9274 | No |
| MV | 4.110 (2.746-6.151) | <0.0001 | Yes |
| ECMO | 1.508 (1.195-1.902) | 0.0005 | Yes |
| Septic_Shock | 2.015 (1.636-2.480) | <0.0001 | Yes |
| MODS | 1.474 (1.165-1.865) | 0.0012 | Yes |
| Diabetes | 0.988 (0.799-1.220) | 0.9078 | No |
| Heart_Disease | 0.641 (0.353-1.165) | 0.1447 | No |
| Hypertension | 0.817 (0.678-0.985) | 0.0344 | Yes |
| Diuretic | 1.240 (1.031-1.492) | 0.0224 | Yes |

**Table S2. Results of the proportional hazards assumption test (Schoenfeld residuals) for Albumin-to-Alkaline Phosphatase Ratio (AAPR).**

| Model 1 (Unadjusted) | | | |
| --- | --- | --- | --- |
| Variable | P |  |  |
| Q2 (vs Q1, ref) | 0.0674 |  |  |
| Q3 (vs Q1, ref) | 0.6305 |  |  |
| Q4 (vs Q1, ref) | 0.2518 |  |  |
| GLOBAL | 0.1800 |  |  |
|  |  |  |  |
| Model 2 (Adjusted for age and sex) | | | |
| Variable | P |  |  |
| Q2 (vs Q1, ref) | 0.1062 |  |  |
| Q3 (vs Q1, ref) | 0.2802 |  |  |
| Q4 (vs Q1, ref) | 0.1084 |  |  |
| Age | <0.0001 |  |  |
| Sex | 0.6261 |  |  |
| GLOBAL | <0.0001 |  |  |
|  |  |  |  |
| Model 3 (Fully adjusted) | | | |
| Variable | P |  |  |
| Q2 (vs Q1, ref) | 0.0018 |  |  |
| Q3 (vs Q1, ref) | 0.2952 |  |  |
| Q4 (vs Q1, ref) | 0.6675 |  |  |
| Age | 0.0007 |  |  |
| Diuretic use | 0.0444 |  |  |
| ECMO | 0.0003 |  |  |
| Hypertension | 0.0108 |  |  |
| MODS | 0.9956 |  |  |
| Mechanical ventilation | 0.4188 |  |  |
| Septic shock | 0.0451 |  |  |
| Sex | 0.7071 |  |  |
| Surgery | 0.0396 |  |  |
| Vasopressor use | <0.0001 |  |  |
| GLOBAL | <0.0001 |  |  |

**Table S3. Results of the proportional hazards assumption test (Schoenfeld residuals) for Prognostic Nutritional Index (PNI).**

| Model 1 (Unadjusted) | | | |
| --- | --- | --- | --- |
| Variable | P |  |  |
| Q2 (vs Q1, ref) | 0.0334 |  |  |
| Q3 (vs Q1, ref) | 0.5250 |  |  |
| Q4 (vs Q1, ref) | 0.6358 |  |  |
| GLOBAL | 0.1608 |  |  |
|  |  |  |  |
| Model 2 (Adjusted for age and sex) | | | |
| Variable | P |  |  |
| Age | <0.0001 |  |  |
| Q2 (vs Q1, ref) | 0.0251 |  |  |
| Q3 (vs Q1, ref) | 0.7492 |  |  |
| Q4 (vs Q1, ref) | 0.7342 |  |  |
| Sex | 0.7008 |  |  |
| GLOBAL | 0.0003 |  |  |
|  |  |  |  |
| Model 3 (Fully adjusted) | | | |
| Variable | P |  |  |
| Age | 0.0027 |  |  |
| Diuretic use | 0.0613 |  |  |
| ECMO | 0.0001 |  |  |
| Hypertension | 0.0118 |  |  |
| MODS | 0.9331 |  |  |
| Mechanical ventilation | 0.5123 |  |  |
| Q2 (vs Q1, ref) | 0.1656 |  |  |
| Q3 (vs Q1, ref) | 0.1307 |  |  |
| Q4 (vs Q1, ref) | 0.1530 |  |  |
| Septic shock | 0.0605 |  |  |
| Sex | 0.8274 |  |  |
| Surgery | 0.0404 |  |  |
| Vasopressor use | <0.0001 |  |  |
| GLOBAL | <0.0001 |  |  |

**Table S4. Results of the proportional hazards assumption test (Schoenfeld residuals) for Albumin-Bilirubin Score (ALBI score).**

| Model 1 (Unadjusted) | | | |
| --- | --- | --- | --- |
| Variable | P |  |  |
| Q2 (vs Q1, ref) | 0.2094 |  |  |
| Q3 (vs Q1, ref) | 0.9297 |  |  |
| Q4 (vs Q1, ref) | 0.2422 |  |  |
| GLOBAL | 0.3992 |  |  |
|  |  |  |  |
| Model 2 (Adjusted for age and sex) | | | |
| Variable | P |  |  |
| Q2 (vs Q1, ref) | 0.0964 |  |  |
| Q3 (vs Q1, ref) | 0.8195 |  |  |
| Q4 (vs Q1, ref) | 0.1285 |  |  |
| Age | <0.0001 |  |  |
| Sex | 0.5735 |  |  |
| GLOBAL | <0.0001 |  |  |
|  |  |  |  |
| Model 3 (Fully adjusted) | | | |
| Variable | P |  |  |
| Q2 (vs Q1, ref) | 0.5295 |  |  |
| Q3 (vs Q1, ref) | 0.2920 |  |  |
| Q4 (vs Q1, ref) | 0.4873 |  |  |
| Age | 0.0009 |  |  |
| Diuretic use | 0.0965 |  |  |
| ECMO | 0.0003 |  |  |
| Hypertension | 0.0111 |  |  |
| MODS | 0.9507 |  |  |
| Mechanical ventilation | 0.4504 |  |  |
| Septic shock | 0.0689 |  |  |
| Sex | 0.6642 |  |  |
| Surgery | 0.0780 |  |  |
| Vasopressor use | <0.0001 |  |  |
| GLOBAL | <0.0001 |  |  |

**Table S5. Results of the proportional hazards assumption test (Schoenfeld residuals) for Blood Urea Nitrogen-to-Creatinine Ratio (BUN/Cr).**

| Model 1 (Unadjusted) | | | |
| --- | --- | --- | --- |
| Variable | P |  |  |
| Q2 (vs Q1, ref) | 0.6208 |  |  |
| Q3 (vs Q1, ref) | 0.9723 |  |  |
| Q4 (vs Q1, ref) | 0.3223 |  |  |
| GLOBAL | 0.7469 |  |  |
|  |  |  |  |
| Model 2 (Adjusted for age and sex) | | | |
| Variable | P |  |  |
| Age | <0.0001 |  |  |
| Q2 (vs Q1, ref) | 0.2651 |  |  |
| Q3 (vs Q1, ref) | 0.4056 |  |  |
| Q4 (vs Q1, ref) | 0.0207 |  |  |
| Sex | 0.8659 |  |  |
| GLOBAL | <0.0001 |  |  |
|  |  |  |  |
| Model 3 (Fully adjusted) | | | |
| Variable | P |  |  |
| Age | 0.0003 |  |  |
| Q2 (vs Q1, ref) | 0.3373 |  |  |
| Q3 (vs Q1, ref) | 0.4108 |  |  |
| Q4 (vs Q1, ref) | 0.1250 |  |  |
| Diuretic use | 0.1258 |  |  |
| ECMO | 0.0004 |  |  |
| Hypertension | 0.0218 |  |  |
| MODS | 0.9957 |  |  |
| Mechanical ventilation | 0.3927 |  |  |
| Septic shock | 0.0445 |  |  |
| Sex | 0.9243 |  |  |
| Surgery | 0.0620 |  |  |
| Vasopressor use | <0.0001 |  |  |
| GLOBAL | <0.0001 |  |  |

**Table S6. Results of the proportional hazards assumption test (Schoenfeld residuals) for Systemic Immune-Inflammation Index (SII).**

| Model 1 (Unadjusted) | | | |
| --- | --- | --- | --- |
| Variable | P |  |  |
| Q2 (vs Q1, ref) | <0.0001 |  |  |
| Q3 (vs Q1, ref) | 0.0824 |  |  |
| Q4 (vs Q1, ref) | 0.0002 |  |  |
| GLOBAL | <0.0001 |  |  |
|  |  |  |  |
| Model 2 (Adjusted for age and sex) | | | |
| Variable | P |  |  |
| Age | <0.0001 |  |  |
| Q2 (vs Q1, ref) | <0.0001 |  |  |
| Q3 (vs Q1, ref) | 0.0784 |  |  |
| Q4 (vs Q1, ref) | 0.0001 |  |  |
| Sex | 0.6085 |  |  |
| GLOBAL | <0.0001 |  |  |
|  |  |  |  |
| Model 3 (Fully adjusted) | | | |
| Variable | P |  |  |
| Age | 0.0007 |  |  |
| Diuretic use | 0.0381 |  |  |
| ECMO | 0.0012 |  |  |
| Hypertension | 0.0225 |  |  |
| MODS | 0.9554 |  |  |
| Mechanical ventilation | 0.4314 |  |  |
| Q2 (vs Q1, ref) | <0.0001 |  |  |
| Q3 (vs Q1, ref) | 0.2299 |  |  |
| Q4 (vs Q1, ref) | 0.0003 |  |  |
| Septic shock | 0.0353 |  |  |
| Sex | 0.7737 |  |  |
| Surgery | 0.0594 |  |  |
| Vasopressor use | <0.0001 |  |  |
| GLOBAL | <0.0001 |  |  |

**Table S7. Results of the proportional hazards assumption test (Schoenfeld residuals) for Neutrophil-to-Lymphocyte Ratio (NLR).**

| Model 1 (Unadjusted) | | | |
| --- | --- | --- | --- |
| Variable | P |  |  |
| Q2 (vs Q1, ref) | 0.1231 |  |  |
| Q3 (vs Q1, ref) | 0.0516 |  |  |
| Q4 (vs Q1, ref) | 0.0017 |  |  |
| GLOBAL | 0.0012 |  |  |
|  |  |  |  |
| Model 2 (Adjusted for age and sex) | | | |
| Variable | P |  |  |
| Age | <0.0001 |  |  |
| Q2 (vs Q1, ref) | 0.1520 |  |  |
| Q3 (vs Q1, ref) | 0.0482 |  |  |
| Q4 (vs Q1, ref) | 0.0032 |  |  |
| Sex | 0.5828 |  |  |
| GLOBAL | <0.0001 |  |  |
|  |  |  |  |
| Model 3 (Fully adjusted) | | | |
| Variable | P |  |  |
| Age | 0.0012 |  |  |
| Diuretic use | 0.0624 |  |  |
| ECMO | 0.0008 |  |  |
| Hypertension | 0.0099 |  |  |
| MODS | 0.8582 |  |  |
| Mechanical ventilation | 0.6341 |  |  |
| Q2 (vs Q1, ref) | 0.0840 |  |  |
| Q3 (vs Q1, ref) | 0.0147 |  |  |
| Q4 (vs Q1, ref) | 0.0003 |  |  |
| Septic shock | 0.0479 |  |  |
| Sex | 0.7564 |  |  |
| Surgery | 0.0513 |  |  |
| Vasopressor use | <0.0001 |  |  |
| GLOBAL | <0.0001 |  |  |

**Table S8. Re-assessment of the Proportional Hazards Assumption After Stratification.**

| **Derived Index** | **Variable** | **Model 1 P** | **Model 2 P** | **Model 3 P** |
| --- | --- | --- | --- | --- |
| AAPR | AAPR_2 | 0.0674 | 0.4411 | 0.1523 |
| AAPR | AAPR_3 | 0.6305 | 0.2814 | 0.5438 |
| AAPR | AAPR_4 | 0.2518 | 0.0547 | 0.2183 |
| AAPR | Sex | - | 0.2567 | 0.0137 |
| AAPR | Diuretic | - | - | 0.4968 |
| AAPR | ECMO | - | - | 0.8984 |
| AAPR | Hypertension | - | - | 0.7950 |
| AAPR | MODS | - | - | 0.0029 |
| AAPR | MV | - | - | 0.0906 |
| AAPR | Septic_Shock | - | - | 0.1184 |
| AAPR | GLOBAL | 0.1800 | 0.1507 | 0.0060 |
| PNI | PNI_2 | 0.0334 | 0.3974 | 0.8025 |
| PNI | PNI_3 | 0.5250 | 0.3082 | 0.5013 |
| PNI | PNI_4 | 0.6358 | 0.8391 | 0.5749 |
| PNI | Sex | - | 0.2349 | 0.0146 |
| PNI | Diuretic | - | - | 0.5219 |
| PNI | ECMO | - | - | 0.9211 |
| PNI | Hypertension | - | - | 0.9309 |
| PNI | MODS | - | - | 0.0049 |
| PNI | MV | - | - | 0.0632 |
| PNI | Septic_Shock | - | - | 0.0453 |
| PNI | GLOBAL | 0.1608 | 0.5239 | 0.0123 |
| ALBI_score | ALBI_score_2 | 0.2094 | 0.1040 | 0.2326 |
| ALBI_score | ALBI_score_3 | 0.9297 | 0.5917 | 0.7090 |
| ALBI_score | ALBI_score_4 | 0.2422 | 0.7809 | 0.4258 |
| ALBI_score | Sex | - | 0.2852 | 0.0122 |
| ALBI_score | Diuretic | - | - | 0.4767 |
| ALBI_score | ECMO | - | - | 0.9133 |
| ALBI_score | Hypertension | - | - | 0.7998 |
| ALBI_score | MODS | - | - | 0.0042 |
| ALBI_score | MV | - | - | 0.0654 |
| ALBI_score | Septic_Shock | - | - | 0.0397 |
| ALBI_score | GLOBAL | 0.3992 | 0.3860 | 0.0056 |
| BUN_Cr | BUN_Cr_2 | 0.6208 | 0.9450 | 0.7879 |
| BUN_Cr | BUN_Cr_3 | 0.9723 | 0.4603 | 0.4220 |
| BUN_Cr | BUN_Cr_4 | 0.3223 | 0.2065 | 0.8194 |
| BUN_Cr | Sex | - | 0.1361 | 0.0063 |
| BUN_Cr | Diuretic | - | - | 0.4080 |
| BUN_Cr | ECMO | - | - | 0.8078 |
| BUN_Cr | Hypertension | - | - | 0.9865 |
| BUN_Cr | MODS | - | - | 0.0025 |
| BUN_Cr | MV | - | - | 0.0702 |
| BUN_Cr | Septic_Shock | - | - | 0.0789 |
| BUN_Cr | GLOBAL | 0.7469 | 0.3586 | 0.0065 |
| SII | SII_2 | <0.0001 | 0.0171 | 0.9409 |
| SII | SII_3 | 0.0824 | 0.0790 | 0.3473 |
| SII | SII_4 | 0.0002 | 0.0161 | 0.6637 |
| SII | Sex | - | 0.2443 | 0.0074 |
| SII | Diuretic | - | - | 0.4184 |
| SII | ECMO | - | - | 0.8156 |
| SII | Hypertension | - | - | 0.8740 |
| SII | MODS | - | - | 0.0024 |
| SII | MV | - | - | 0.0745 |
| SII | Septic_Shock | - | - | 0.0895 |
| SII | GLOBAL | <0.0001 | 0.0031 | 0.0069 |
| NLR | NLR_2 | 0.1231 | 0.9310 | 0.5017 |
| NLR | NLR_3 | 0.0516 | 0.5289 | 0.7773 |
| NLR | NLR_4 | 0.0017 | 0.1685 | 0.9471 |
| NLR | Sex | - | 0.2287 | 0.0077 |
| NLR | Diuretic | - | - | 0.4180 |
| NLR | ECMO | - | - | 0.7965 |
| NLR | Hypertension | - | - | 0.9253 |
| NLR | MODS | - | - | 0.0025 |
| NLR | MV | - | - | 0.0565 |
| NLR | Septic_Shock | - | - | 0.0576 |
| NLR | GLOBAL | 0.0012 | 0.4411 | 0.0058 |

**Table S9. Sensitivity Analysis Stratified by Median Event Time.**

| **Analysis** | **N** | **Events** | **AAPR Q4 vs Q1, HR (95% CI)** | **P value** | **P for trend** |
| --- | --- | --- | --- | --- | --- |
| Main model (full follow-up) | 1732 | 507 | 0.683 (0.524–0.890) | 0.0047 | 0.0073 |
| Early period (0–10 days) | 1732 | 268 | 0.760 (0.526–1.097) | 0.1431 | 0.1050 |
| Late period (>10 days) | 1084 | 239 | 0.546 (0.372–0.801) | 0.0020 | 0.0119 |

| Derived Indices | Threshold | AIC | HR_below | CI_below | P_below | HR_above | CI_above | P_above |
| --- | --- | --- | --- | --- | --- | --- | --- | --- |
| AAPR | 0.2028 | 4680.8 | 0.001 | (0.000-0.017) | <0.0001 | 0.797 | (0.475-1.340) | 0.3928 |
| PNI | 33.8 | 4696.38 | 0.966 | (0.944-0.989) | 0.0043 | 1.022 | (1.005-1.039) | 0.0128 |
| ALBI_score | -2.7049 | 4688.19 | 0.122 | (0.051-0.289) | <0.0001 | 1.178 | (0.994-1.396) | 0.0592 |
| BUN_Cr | 30.5147 | 4702.44 | 1.004 | (0.991-1.018) | 0.5478 | 1.015 | (0.995-1.036) | 0.1483 |
| SII | 1169.2718 | 4701.43 | 1.000 | (0.999-1.000) | 0.1251 | 1.000 | (1.000-1.000) | 0.0403 |
| NLR | 12 | 4698.82 | 0.970 | (0.941-1.000) | 0.0523 | 1.005 | (1.001-1.009) | 0.0080 |

**Table S10. Threshold Effect Analysis of Derived Indices on In-Hospital Mortality Using Two-Piecewise Linear Cox Regression Models**

**Table S11. Subgroup Analyses of the Association Between Derived Indices and In-Hospital Mortality with Adaptive Dichotomization Based on Restricted Cubic Spline Results.**

| **Derived Index** | **Subgroup** | **Dichotomization** | **Cutoff** | **N** | **Events** | **HR** | **95%CI_lo** | **95%CI_hi** | **P value** | **P for interaction** |
| --- | --- | --- | --- | --- | --- | --- | --- | --- | --- | --- |
| AAPR | Age < 65 | RCS Threshold | 0.203 | 1121 | 295 | 0.41 | 0.30 | 0.56 | <0.001 | 0.002 |
| AAPR | Age ≥ 65 | RCS Threshold | 0.203 | 611 | 212 | 0.69 | 0.48 | 0.98 | 0.037 |  |
| AAPR | Male | RCS Threshold | 0.203 | 508 | 143 | 0.49 | 0.32 | 0.76 | 0.001 | 0.899 |
| AAPR | Female | RCS Threshold | 0.203 | 1224 | 364 | 0.49 | 0.37 | 0.64 | <0.001 |  |
| AAPR | HTN = Yes | RCS Threshold | 0.203 | 629 | 161 | 0.74 | 0.39 | 1.40 | 0.347 | 0.179 |
| AAPR | HTN = No | RCS Threshold | 0.203 | 1103 | 346 | 0.47 | 0.37 | 0.61 | <0.001 |  |
| AAPR | Septic Shock = Yes | RCS Threshold | 0.203 | 232 | 115 | 0.66 | 0.44 | 1.00 | 0.051 | 0.233 |
| AAPR | Septic Shock = No | RCS Threshold | 0.203 | 1500 | 392 | 0.50 | 0.38 | 0.66 | <0.001 |  |
| AAPR | MODS = Yes | RCS Threshold | 0.203 | 220 | 83 | 0.47 | 0.30 | 0.74 | 0.001 | 0.454 |
| AAPR | MODS = No | RCS Threshold | 0.203 | 1512 | 424 | 0.54 | 0.41 | 0.71 | <0.001 |  |
| AAPR | MV = Yes | RCS Threshold | 0.203 | 1334 | 482 | 0.53 | 0.41 | 0.67 | <0.001 | 0.135 |
| AAPR | MV = No | RCS Threshold | 0.203 | 398 | 25 | 0.25 | 0.10 | 0.64 | 0.003 |  |
| AAPR | Surgery = Yes | RCS Threshold | 0.203 | 901 | 261 | 0.73 | 0.47 | 1.16 | 0.186 | 0.060 |
| AAPR | Surgery = No | RCS Threshold | 0.203 | 831 | 246 | 0.45 | 0.34 | 0.60 | <0.001 |  |
| AAPR | ECMO = Yes | RCS Threshold | 0.203 | 186 | 86 | 0.41 | 0.18 | 0.95 | 0.038 | 0.884 |
| AAPR | ECMO = No | RCS Threshold | 0.203 | 1546 | 421 | 0.47 | 0.37 | 0.60 | <0.001 |  |
| AAPR | Vasopressor = Yes | RCS Threshold | 0.203 | 708 | 251 | 0.51 | 0.37 | 0.69 | <0.001 | 0.711 |
| AAPR | Vasopressor = No | RCS Threshold | 0.203 | 1024 | 256 | 0.51 | 0.36 | 0.73 | <0.001 |  |
| AAPR | Diuretic = Yes | RCS Threshold | 0.203 | 1092 | 338 | 0.51 | 0.39 | 0.66 | <0.001 | 0.953 |
| AAPR | Diuretic = No | RCS Threshold | 0.203 | 640 | 169 | 0.47 | 0.30 | 0.74 | 0.001 |  |
| PNI | Age < 65 | RCS Threshold | 33.800 | 1121 | 295 | 0.91 | 0.72 | 1.15 | 0.419 | 0.161 |
| PNI | Age ≥ 65 | RCS Threshold | 33.800 | 611 | 212 | 0.81 | 0.62 | 1.06 | 0.128 |  |
| PNI | Male | RCS Threshold | 33.800 | 508 | 143 | 0.89 | 0.64 | 1.24 | 0.502 | 0.567 |
| PNI | Female | RCS Threshold | 33.800 | 1224 | 364 | 0.80 | 0.65 | 0.99 | 0.038 |  |
| PNI | HTN = Yes | RCS Threshold | 33.800 | 629 | 161 | 1.50 | 1.05 | 2.14 | 0.025 | <0.001 |
| PNI | HTN = No | RCS Threshold | 33.800 | 1103 | 346 | 0.68 | 0.55 | 0.84 | <0.001 |  |
| PNI | Septic Shock = Yes | RCS Threshold | 33.800 | 232 | 115 | 0.83 | 0.57 | 1.22 | 0.339 | 0.637 |
| PNI | Septic Shock = No | RCS Threshold | 33.800 | 1500 | 392 | 0.92 | 0.75 | 1.12 | 0.400 |  |
| PNI | MODS = Yes | RCS Threshold | 33.800 | 220 | 83 | 0.74 | 0.48 | 1.15 | 0.180 | 0.464 |
| PNI | MODS = No | RCS Threshold | 33.800 | 1512 | 424 | 0.87 | 0.72 | 1.06 | 0.168 |  |
| PNI | MV = Yes | RCS Threshold | 33.800 | 1334 | 482 | 0.87 | 0.73 | 1.04 | 0.125 | 0.384 |
| PNI | MV = No | RCS Threshold | 33.800 | 398 | 25 | 1.32 | 0.53 | 3.32 | 0.549 |  |
| PNI | Surgery = Yes | RCS Threshold | 33.800 | 901 | 261 | 0.90 | 0.70 | 1.15 | 0.409 | 0.602 |
| PNI | Surgery = No | RCS Threshold | 33.800 | 831 | 246 | 0.80 | 0.62 | 1.03 | 0.084 |  |
| PNI | ECMO = Yes | RCS Threshold | 33.800 | 186 | 86 | 0.81 | 0.52 | 1.27 | 0.363 | 0.870 |
| PNI | ECMO = No | RCS Threshold | 33.800 | 1546 | 421 | 0.80 | 0.66 | 0.97 | 0.020 |  |
| PNI | Vasopressor = Yes | RCS Threshold | 33.800 | 708 | 251 | 0.81 | 0.63 | 1.04 | 0.091 | 0.942 |
| PNI | Vasopressor = No | RCS Threshold | 33.800 | 1024 | 256 | 0.85 | 0.66 | 1.08 | 0.181 |  |
| PNI | Diuretic = Yes | RCS Threshold | 33.800 | 1092 | 338 | 0.89 | 0.72 | 1.10 | 0.285 | 0.246 |
| PNI | Diuretic = No | RCS Threshold | 33.800 | 640 | 169 | 0.73 | 0.54 | 0.98 | 0.039 |  |
| ALBI_score | Age < 65 | RCS Threshold | -2.705 | 1121 | 295 | 0.95 | 0.65 | 1.38 | 0.775 | 0.810 |
| ALBI_score | Age ≥ 65 | RCS Threshold | -2.705 | 611 | 212 | 1.01 | 0.47 | 2.15 | 0.983 |  |
| ALBI_score | Male | RCS Threshold | -2.705 | 508 | 143 | 0.92 | 0.48 | 1.75 | 0.793 | 0.746 |
| ALBI_score | Female | RCS Threshold | -2.705 | 1224 | 364 | 1.06 | 0.72 | 1.56 | 0.767 |  |
| ALBI_score | HTN = Yes | RCS Threshold | -2.705 | 629 | 161 | 0.66 | 0.42 | 1.04 | 0.075 | 0.104 |
| ALBI_score | HTN = No | RCS Threshold | -2.705 | 1103 | 346 | 1.34 | 0.81 | 2.21 | 0.253 |  |
| ALBI_score | Septic Shock = Yes | RCS Threshold | -2.705 | 232 | 115 | 1.46 | 0.20 | 10.49 | 0.705 | 0.619 |
| ALBI_score | Septic Shock = No | RCS Threshold | -2.705 | 1500 | 392 | 0.91 | 0.65 | 1.28 | 0.581 |  |
| ALBI_score | MODS = Yes | RCS Threshold | -2.705 | 220 | 83 | 1.37 | 0.34 | 5.56 | 0.663 | 0.654 |
| ALBI_score | MODS = No | RCS Threshold | -2.705 | 1512 | 424 | 0.97 | 0.69 | 1.36 | 0.855 |  |
| ALBI_score | MV = Yes | RCS Threshold | -2.705 | 1334 | 482 | 0.92 | 0.65 | 1.31 | 0.652 | 0.558 |
| ALBI_score | MV = No | RCS Threshold | -2.705 | 398 | 25 | 0.66 | 0.24 | 1.76 | 0.403 |  |
| ALBI_score | Surgery = Yes | RCS Threshold | -2.705 | 901 | 261 | 0.79 | 0.53 | 1.18 | 0.251 | 0.199 |
| ALBI_score | Surgery = No | RCS Threshold | -2.705 | 831 | 246 | 1.36 | 0.74 | 2.49 | 0.320 |  |
| ALBI_score | ECMO = Yes | RCS Threshold | -2.705 | 186 | 86 | 1.31 | 0.53 | 3.24 | 0.561 | 0.499 |
| ALBI_score | ECMO = No | RCS Threshold | -2.705 | 1546 | 421 | 1.00 | 0.70 | 1.43 | 0.992 |  |
| ALBI_score | Vasopressor = Yes | RCS Threshold | -2.705 | 708 | 251 | 0.84 | 0.53 | 1.34 | 0.469 | 0.256 |
| ALBI_score | Vasopressor = No | RCS Threshold | -2.705 | 1024 | 256 | 1.10 | 0.69 | 1.75 | 0.699 |  |
| ALBI_score | Diuretic = Yes | RCS Threshold | -2.705 | 1092 | 338 | 1.17 | 0.73 | 1.86 | 0.511 | 0.427 |
| ALBI_score | Diuretic = No | RCS Threshold | -2.705 | 640 | 169 | 0.83 | 0.52 | 1.35 | 0.456 |  |
| BUN_Cr | Age < 65 | Median | 16.220 | 1121 | 295 | 1.19 | 0.94 | 1.49 | 0.141 | 0.416 |
| BUN_Cr | Age ≥ 65 | Median | 16.220 | 611 | 212 | 1.14 | 0.86 | 1.52 | 0.373 |  |
| BUN_Cr | Male | Median | 16.220 | 508 | 143 | 1.09 | 0.77 | 1.53 | 0.624 | 0.326 |
| BUN_Cr | Female | Median | 16.220 | 1224 | 364 | 1.32 | 1.07 | 1.62 | 0.009 |  |
| BUN_Cr | HTN = Yes | Median | 16.220 | 629 | 161 | 1.15 | 0.84 | 1.57 | 0.383 | 0.992 |
| BUN_Cr | HTN = No | Median | 16.220 | 1103 | 346 | 1.23 | 0.99 | 1.52 | 0.065 |  |
| BUN_Cr | Septic Shock = Yes | Median | 16.220 | 232 | 115 | 1.10 | 0.74 | 1.62 | 0.641 | 0.689 |
| BUN_Cr | Septic Shock = No | Median | 16.220 | 1500 | 392 | 1.21 | 0.99 | 1.48 | 0.060 |  |
| BUN_Cr | MODS = Yes | Median | 16.220 | 220 | 83 | 2.00 | 1.27 | 3.14 | 0.003 | 0.048 |
| BUN_Cr | MODS = No | Median | 16.220 | 1512 | 424 | 1.16 | 0.95 | 1.40 | 0.139 |  |
| BUN_Cr | MV = Yes | Median | 16.220 | 1334 | 482 | 1.17 | 0.97 | 1.40 | 0.098 | 0.404 |
| BUN_Cr | MV = No | Median | 16.220 | 398 | 25 | 1.59 | 0.72 | 3.53 | 0.249 |  |
| BUN_Cr | Surgery = Yes | Median | 16.220 | 901 | 261 | 1.26 | 0.98 | 1.61 | 0.068 | 0.681 |
| BUN_Cr | Surgery = No | Median | 16.220 | 831 | 246 | 1.20 | 0.93 | 1.54 | 0.166 |  |
| BUN_Cr | ECMO = Yes | Median | 16.220 | 186 | 86 | 0.96 | 0.62 | 1.47 | 0.843 | 0.328 |
| BUN_Cr | ECMO = No | Median | 16.220 | 1546 | 421 | 1.29 | 1.06 | 1.56 | 0.010 |  |
| BUN_Cr | Vasopressor = Yes | Median | 16.220 | 708 | 251 | 1.10 | 0.86 | 1.42 | 0.453 | 0.297 |
| BUN_Cr | Vasopressor = No | Median | 16.220 | 1024 | 256 | 1.28 | 1.00 | 1.64 | 0.048 |  |
| BUN_Cr | Diuretic = Yes | Median | 16.220 | 1092 | 338 | 1.22 | 0.98 | 1.52 | 0.072 | 0.922 |
| BUN_Cr | Diuretic = No | Median | 16.220 | 640 | 169 | 1.22 | 0.90 | 1.65 | 0.192 |  |
| SII | Age < 65 | Median | 2085.887 | 1121 | 295 | 0.99 | 0.79 | 1.24 | 0.917 | 0.257 |
| SII | Age ≥ 65 | Median | 2085.887 | 611 | 212 | 1.11 | 0.85 | 1.45 | 0.446 |  |
| SII | Male | Median | 2085.887 | 508 | 143 | 0.94 | 0.68 | 1.30 | 0.711 | 0.520 |
| SII | Female | Median | 2085.887 | 1224 | 364 | 1.07 | 0.87 | 1.32 | 0.504 |  |
| SII | HTN = Yes | Median | 2085.887 | 629 | 161 | 0.88 | 0.65 | 1.20 | 0.433 | 0.169 |
| SII | HTN = No | Median | 2085.887 | 1103 | 346 | 1.12 | 0.91 | 1.38 | 0.291 |  |
| SII | Septic Shock = Yes | Median | 2085.887 | 232 | 115 | 1.37 | 0.95 | 1.98 | 0.096 | 0.147 |
| SII | Septic Shock = No | Median | 2085.887 | 1500 | 392 | 1.00 | 0.82 | 1.22 | 0.987 |  |
| SII | MODS = Yes | Median | 2085.887 | 220 | 83 | 1.16 | 0.75 | 1.78 | 0.511 | 0.553 |
| SII | MODS = No | Median | 2085.887 | 1512 | 424 | 1.01 | 0.84 | 1.23 | 0.892 |  |
| SII | MV = Yes | Median | 2085.887 | 1334 | 482 | 1.02 | 0.85 | 1.22 | 0.859 | 0.510 |
| SII | MV = No | Median | 2085.887 | 398 | 25 | 1.37 | 0.62 | 3.02 | 0.435 |  |
| SII | Surgery = Yes | Median | 2085.887 | 901 | 261 | 0.98 | 0.77 | 1.25 | 0.877 | 0.473 |
| SII | Surgery = No | Median | 2085.887 | 831 | 246 | 1.10 | 0.86 | 1.41 | 0.452 |  |
| SII | ECMO = Yes | Median | 2085.887 | 186 | 86 | 0.61 | 0.40 | 0.94 | 0.026 | 0.067 |
| SII | ECMO = No | Median | 2085.887 | 1546 | 421 | 1.08 | 0.89 | 1.30 | 0.447 |  |
| SII | Vasopressor = Yes | Median | 2085.887 | 708 | 251 | 1.19 | 0.93 | 1.52 | 0.172 | 0.150 |
| SII | Vasopressor = No | Median | 2085.887 | 1024 | 256 | 0.95 | 0.74 | 1.21 | 0.672 |  |
| SII | Diuretic = Yes | Median | 2085.887 | 1092 | 338 | 1.12 | 0.91 | 1.39 | 0.294 | 0.197 |
| SII | Diuretic = No | Median | 2085.887 | 640 | 169 | 0.88 | 0.65 | 1.19 | 0.417 |  |
| NLR | Age < 65 | Median | 13.293 | 1121 | 295 | 1.03 | 0.82 | 1.29 | 0.822 | 0.693 |
| NLR | Age ≥ 65 | Median | 13.293 | 611 | 212 | 1.05 | 0.80 | 1.38 | 0.714 |  |
| NLR | Male | Median | 13.293 | 508 | 143 | 1.03 | 0.74 | 1.43 | 0.855 | 0.940 |
| NLR | Female | Median | 13.293 | 1224 | 364 | 1.05 | 0.85 | 1.29 | 0.664 |  |
| NLR | HTN = Yes | Median | 13.293 | 629 | 161 | 0.91 | 0.67 | 1.24 | 0.552 | 0.338 |
| NLR | HTN = No | Median | 13.293 | 1103 | 346 | 1.10 | 0.89 | 1.36 | 0.384 |  |
| NLR | Septic Shock = Yes | Median | 13.293 | 232 | 115 | 1.03 | 0.71 | 1.50 | 0.858 | 0.914 |
| NLR | Septic Shock = No | Median | 13.293 | 1500 | 392 | 1.00 | 0.82 | 1.22 | 0.964 |  |
| NLR | MODS = Yes | Median | 13.293 | 220 | 83 | 1.24 | 0.79 | 1.94 | 0.351 | 0.332 |
| NLR | MODS = No | Median | 13.293 | 1512 | 424 | 0.99 | 0.82 | 1.19 | 0.885 |  |
| NLR | MV = Yes | Median | 13.293 | 1334 | 482 | 0.97 | 0.81 | 1.16 | 0.753 | 0.544 |
| NLR | MV = No | Median | 13.293 | 398 | 25 | 1.28 | 0.58 | 2.82 | 0.534 |  |
| NLR | Surgery = Yes | Median | 13.293 | 901 | 261 | 0.92 | 0.72 | 1.17 | 0.506 | 0.174 |
| NLR | Surgery = No | Median | 13.293 | 831 | 246 | 1.20 | 0.93 | 1.54 | 0.157 |  |
| NLR | ECMO = Yes | Median | 13.293 | 186 | 86 | 0.76 | 0.49 | 1.16 | 0.204 | 0.293 |
| NLR | ECMO = No | Median | 13.293 | 1546 | 421 | 1.08 | 0.89 | 1.31 | 0.438 |  |
| NLR | Vasopressor = Yes | Median | 13.293 | 708 | 251 | 1.07 | 0.84 | 1.37 | 0.585 | 0.825 |
| NLR | Vasopressor = No | Median | 13.293 | 1024 | 256 | 1.02 | 0.80 | 1.30 | 0.883 |  |
| NLR | Diuretic = Yes | Median | 13.293 | 1092 | 338 | 1.13 | 0.91 | 1.40 | 0.265 | 0.208 |
| NLR | Diuretic = No | Median | 13.293 | 640 | 169 | 0.88 | 0.65 | 1.19 | 0.399 |  |

**Table S12. Performance evaluation metrics of machine learning models on the training set (95% CI).**

| Model | AUC | Sensitivity | Specificity | Precision | NPV | F1 | MCC | Accuracy | Youden's Index |  |
| --- | --- | --- | --- | --- | --- | --- | --- | --- | --- | --- |
| Random Forest | 0.900 (0.882-0.918) | 0.878 (0.844-0.913) | 0.756 (0.728-0.784) | 0.598 (0.554-0.638) | 0.938 (0.919-0.955) | 0.711 (0.677-0.744) | 0.582 (0.540-0.625) | 0.791 (0.769-0.813) | 0.634 (0.589-0.678) |  |
| XGBoost | 0.879 (0.859-0.898) | 0.792 (0.750-0.831) | 0.833 (0.808-0.857) | 0.662 (0.617-0.705) | 0.906 (0.886-0.925) | 0.721 (0.684-0.754) | 0.596 (0.548-0.642) | 0.821 (0.800-0.843) | 0.624 (0.578-0.671) |  |
| LightGBM | 0.877 (0.856-0.896) | 0.831 (0.791-0.869) | 0.779 (0.753-0.806) | 0.609 (0.567-0.652) | 0.918 (0.898-0.937) | 0.703 (0.666-0.735) | 0.567 (0.520-0.613) | 0.794 (0.772-0.817) | 0.610 (0.562-0.658) |  |
| Gradient Boosting | 0.843 (0.820-0.865) | 0.822 (0.784-0.861) | 0.701 (0.669-0.731) | 0.532 (0.490-0.571) | 0.905 (0.882-0.926) | 0.645 (0.610-0.678) | 0.477 (0.430-0.525) | 0.736 (0.712-0.760) | 0.522 (0.475-0.572) |  |
| SVM | 0.734 (0.704-0.761) | 0.791 (0.748-0.834) | 0.567 (0.534-0.601) | 0.430 (0.392-0.468) | 0.868 (0.838-0.895) | 0.557 (0.516-0.594) | 0.326 (0.274-0.378) | 0.632 (0.606-0.660) | 0.358 (0.301-0.414) |  |
| Logistic Regression | 0.731 (0.701-0.757) | 0.777 (0.732-0.820) | 0.583 (0.549-0.618) | 0.435 (0.398-0.474) | 0.863 (0.836-0.891) | 0.558 (0.520-0.595) | 0.328 (0.275-0.379) | 0.640 (0.612-0.666) | 0.360 (0.304-0.414) |  |

**Table S13. Performance evaluation metrics of machine learning models on the test set (95% CI).**

| Model | AUC | Sensitivity | Specificity | Precision | NPV | F1 | MCC | Accuracy | Youden's Index |  |
| --- | --- | --- | --- | --- | --- | --- | --- | --- | --- | --- |
| Gradient Boosting | 0.728 (0.681-0.774) | 0.689 (0.613-0.768) | 0.658 (0.609-0.707) | 0.455 (0.392-0.522) | 0.836 (0.792-0.882) | 0.547 (0.487-0.611) | 0.318 (0.239-0.409) | 0.667 (0.628-0.711) | 0.347 (0.264-0.444) |  |
| Random Forest | 0.722 (0.674-0.768) | 0.664 (0.588-0.736) | 0.674 (0.624-0.723) | 0.457 (0.393-0.529) | 0.829 (0.785-0.870) | 0.541 (0.480-0.602) | 0.311 (0.230-0.393) | 0.671 (0.630-0.713) | 0.337 (0.248-0.427) |  |
| XGBoost | 0.716 (0.665-0.761) | 0.532 (0.453-0.610) | 0.750 (0.706-0.796) | 0.469 (0.392-0.547) | 0.795 (0.753-0.836) | 0.498 (0.423-0.566) | 0.273 (0.182-0.363) | 0.686 (0.645-0.728) | 0.283 (0.187-0.376) |  |
| Logistic Regression | 0.712 (0.664-0.759) | 0.721 (0.653-0.791) | 0.595 (0.544-0.645) | 0.424 (0.364-0.484) | 0.837 (0.794-0.880) | 0.533 (0.477-0.588) | 0.287 (0.209-0.367) | 0.632 (0.590-0.672) | 0.316 (0.229-0.402) |  |
| SVM | 0.711 (0.664-0.757) | 0.721 (0.653-0.791) | 0.567 (0.515-0.619) | 0.408 (0.350-0.466) | 0.831 (0.786-0.875) | 0.521 (0.463-0.578) | 0.262 (0.183-0.339) | 0.612 (0.568-0.655) | 0.288 (0.204-0.371) |  |
| LightGBM | 0.698 (0.646-0.747) | 0.591 (0.512-0.669) | 0.699 (0.652-0.749) | 0.448 (0.378-0.520) | 0.805 (0.762-0.846) | 0.509 (0.444-0.575) | 0.271 (0.182-0.361) | 0.668 (0.626-0.709) | 0.290 (0.194-0.385) |  |

**Table S14. Pairwise comparison of AUC among machine learning models using the DeLong test.**

| Model | AUC | Reference_Model | Reference_AUC | Delta_AUC | Z_statistic | P_value | Significance |
| --- | --- | --- | --- | --- | --- | --- | --- |
| Random Forest | 0.7215 | Gradient Boosting | 0.7281 | 0.0066 | 0.608 | 0.5431 | ns |
| XGBoost | 0.7158 | Gradient Boosting | 0.7281 | 0.0123 | 0.888 | 0.3747 | ns |
| Logistic Regression | 0.7126 | Gradient Boosting | 0.7281 | 0.0155 | 1.103 | 0.2701 | ns |
| SVM | 0.7116 | Gradient Boosting | 0.7281 | 0.0165 | 1.137 | 0.2554 | ns |
| LightGBM | 0.6984 | Gradient Boosting | 0.7281 | 0.0297 | 2.235 | 0.0254 | * |

**Figure S1. Flowchart of Patient Screening and Enrollment**

**
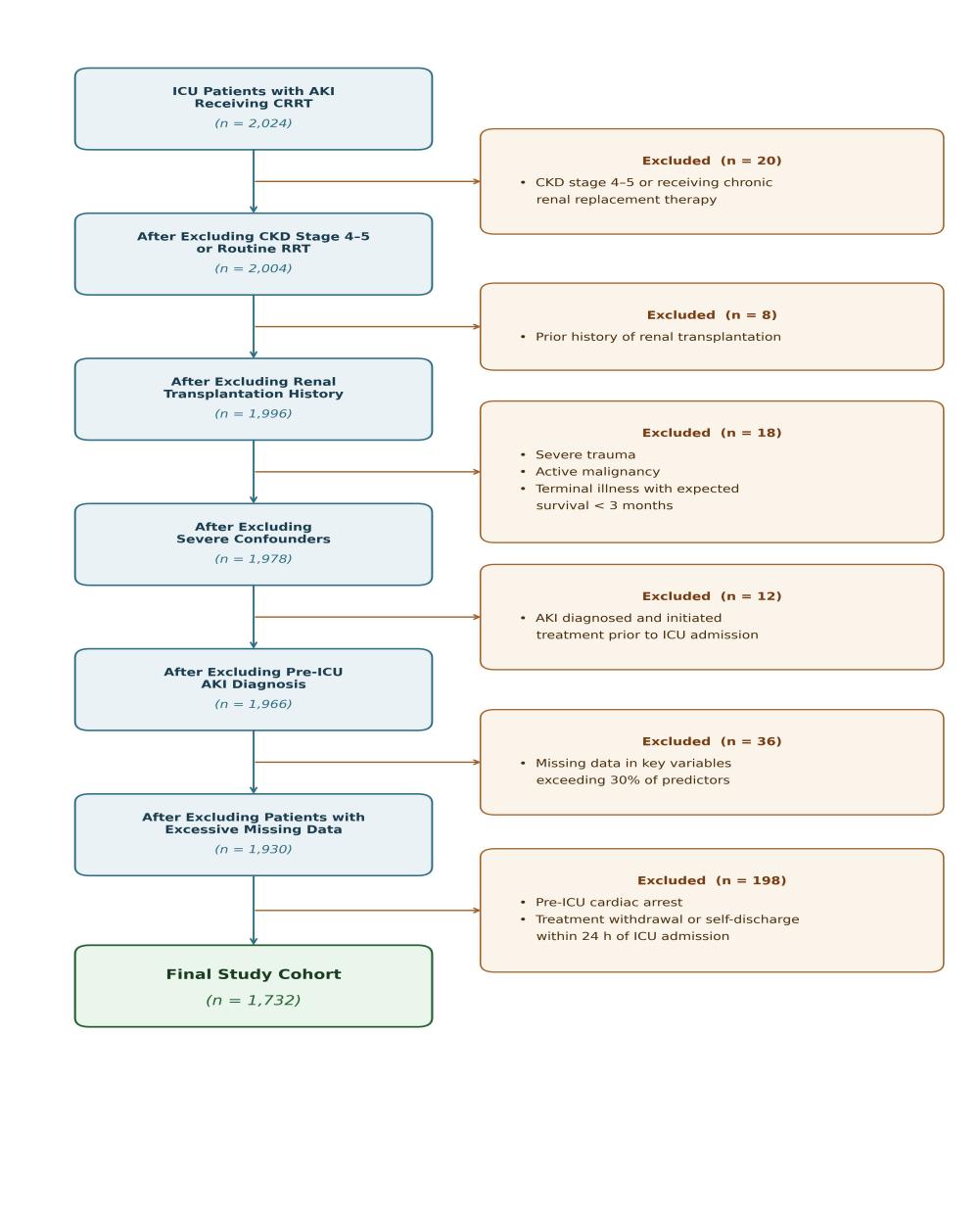
**

**Figure S2. Performance Evaluation of Six Machine Learning Models for Predicting In-Hospital Mortality in AKI-CRRT Patients. (A) Precision-recall curves; (B) Decision curve analysis; (C) Calibration curves on the training set; (D) Calibration curves on the test set.**


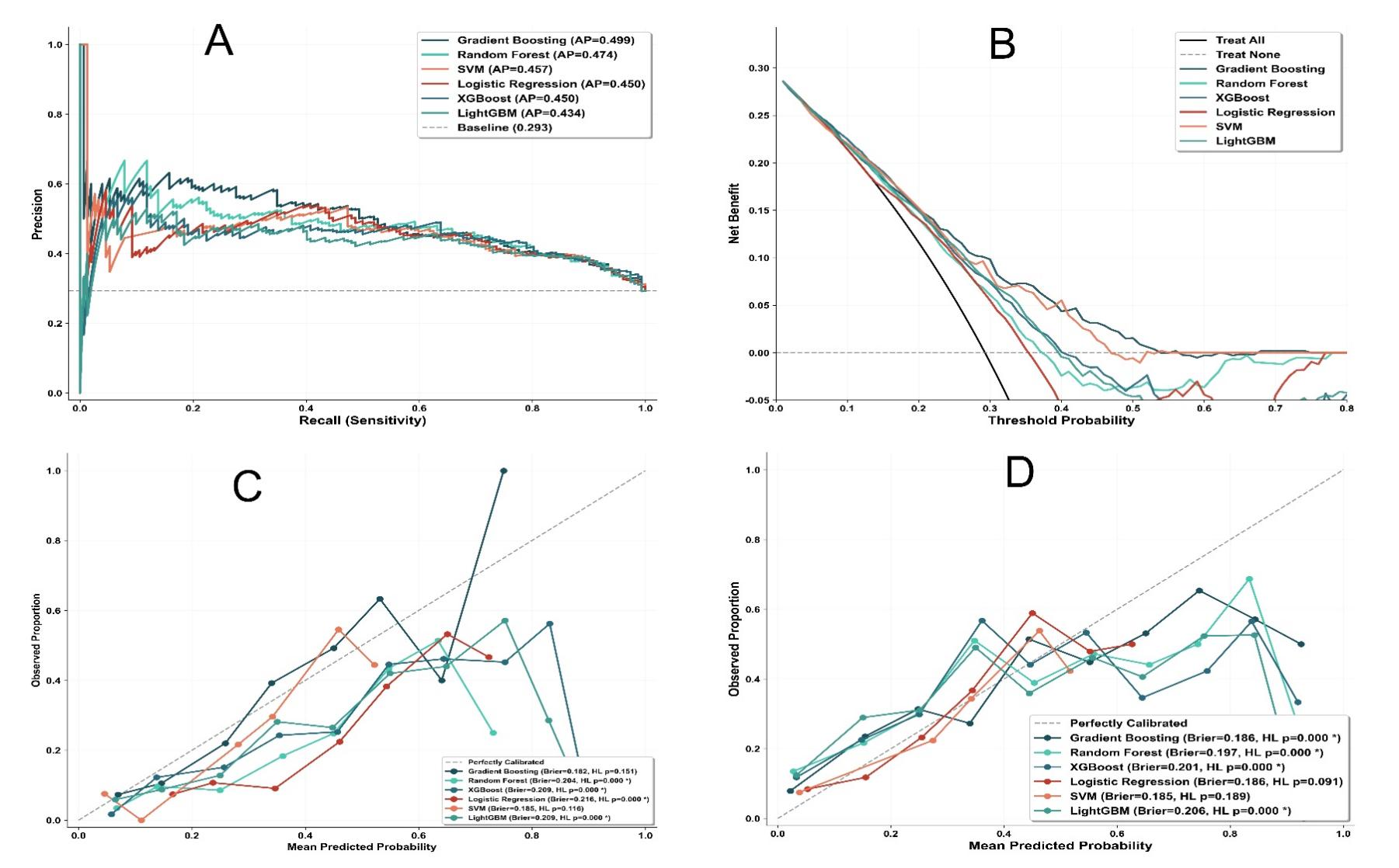


**Figure S3. SHAP Dependence Plots for All 16 Selected Features in the Gradient Boosting Model**


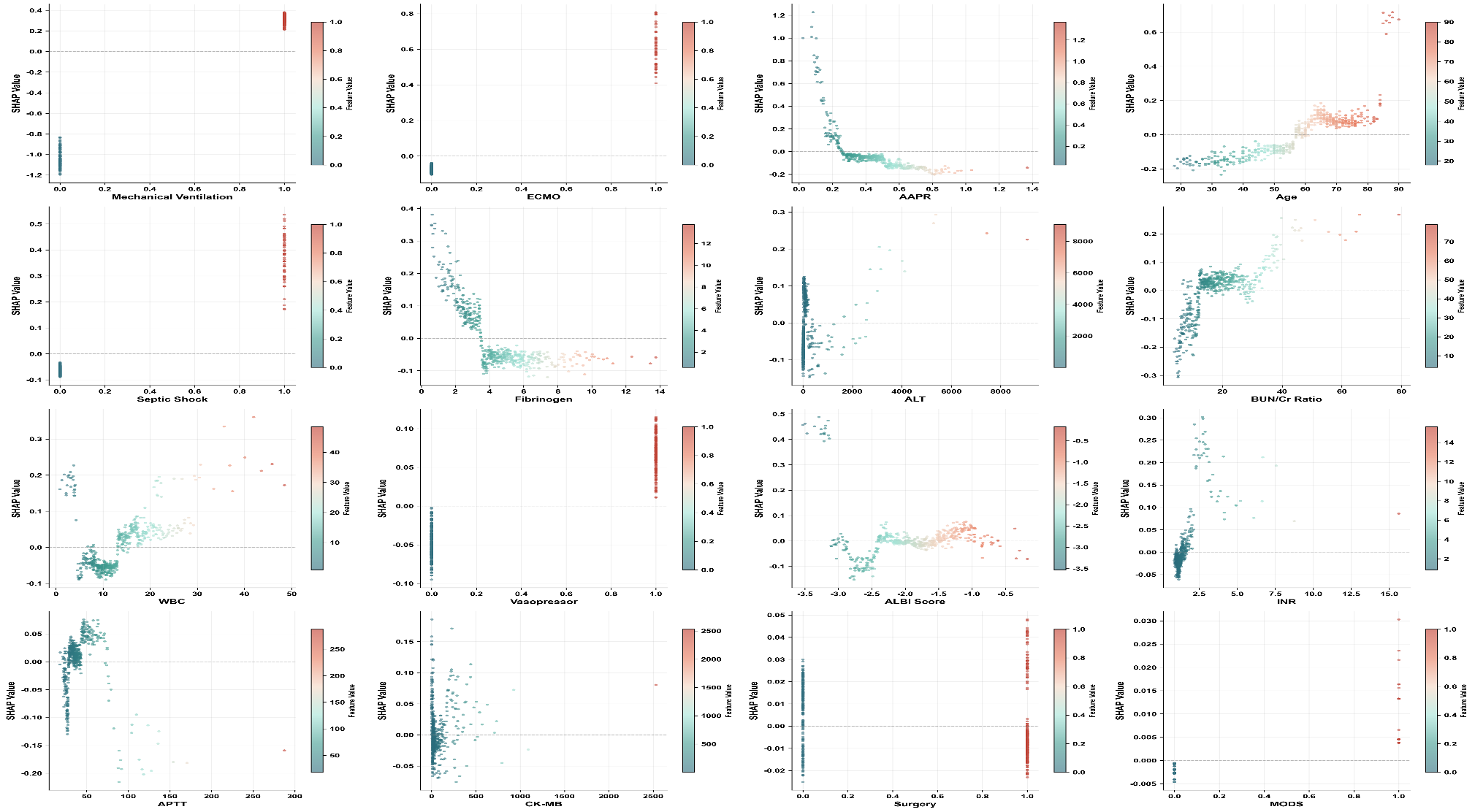


**Figure S4. SHAP Interaction Plots for the Top 5 Most Important Features in the Gradient Boosting Model**


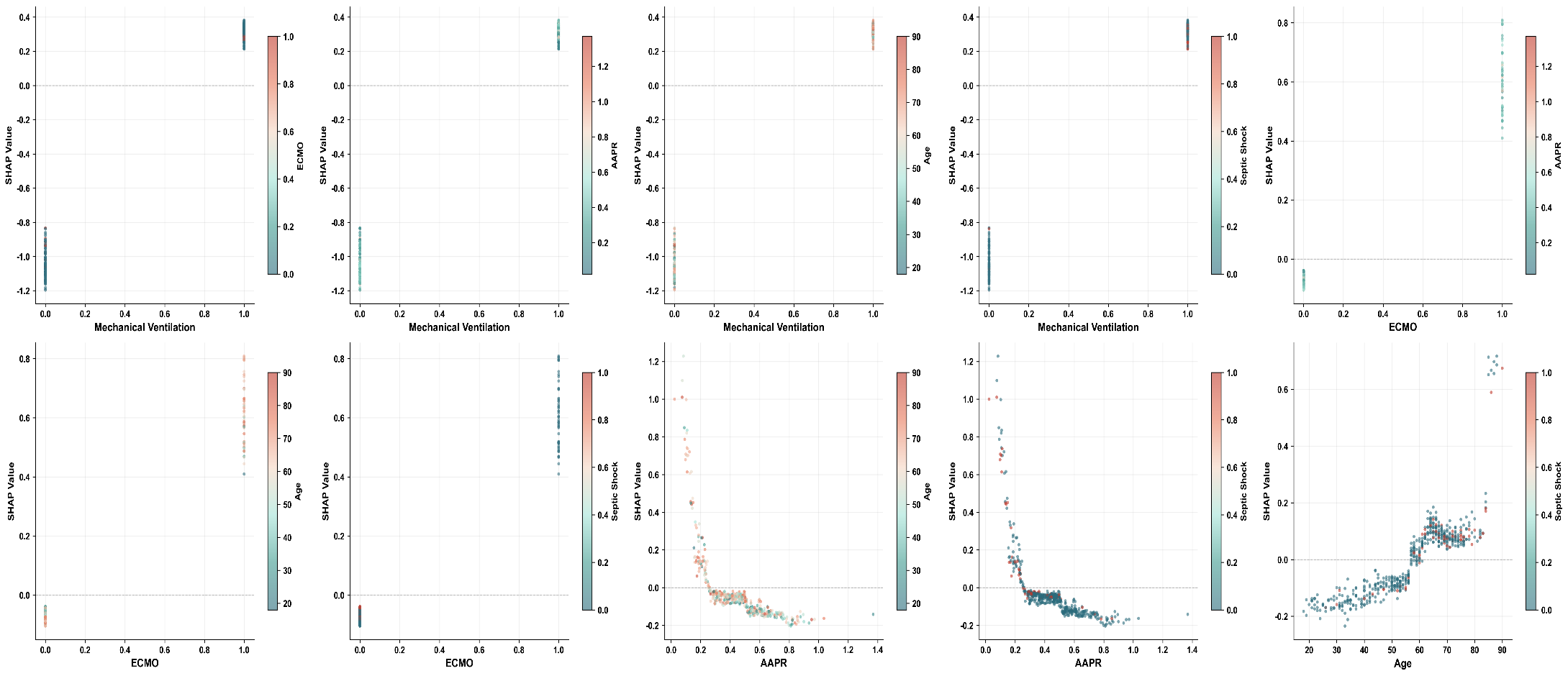


**Figure S5. SHAP Waterfall Plots for Three Representative Individual Patients. (A) A low-risk patient (survivor); (B) A moderate-risk patient; (C) A high-risk patient (non-survivor).**


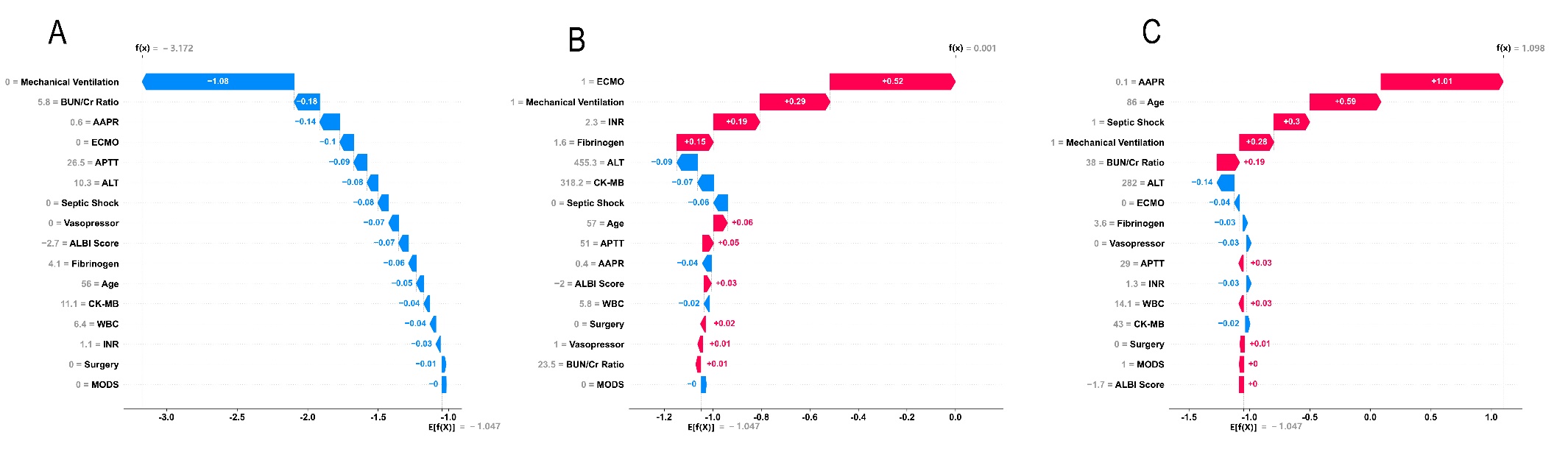

Supplement: Supplementary file 1 [file Table_1.DOCX]
